# Species‐specific roles of cellular communication network proteins in cartilage development: A comparative study using in vitro chondrogenic models

**DOI:** 10.1002/ccs3.70089

**Published:** 2026-06-02

**Authors:** Zhangzheng Wang, Judit Vágó, Roland Takács, Szilárd Póliska, Ee Hyun Kim, Eun‐Jung Jin, Satoshi Kubota, Celina G Kleer, Bernard Perbal, Csaba Matta

**Affiliations:** ^1^ Department of Anatomy, Histology and Embryology Faculty of Medicine University of Debrecen Debrecen Hungary; ^2^ Genomic Medicine and Bioinformatics Core Facility Department of Biochemistry and Molecular Biology Faculty of Medicine University of Debrecen Debrecen Hungary; ^3^ Department of Biomedical Materials Science Graduate School of JABA Wonkwang University Iksan Republic of Korea; ^4^ Integrated Omics Institute Wonkwang University Iksan Republic of Korea; ^5^ Organelle Therapeutics Inc. Jeonbuk South Korea; ^6^ Department of Biochemistry and Molecular Dentistry Okayama University, Faculty of Medicine, Dentistry and Pharmaceutical Sciences Okayama Japan; ^7^ Department of Pathology University of Michigan Medical School Ann Arbor Michigan USA; ^8^ Rogel Cancer Center University of Michigan Ann Arbor Michigan USA; ^9^ International CCN Society Nice France

**Keywords:** cartilage regeneration, CCN, chondrogenesis, osteoarthritis, regulatory network, transcriptomics

## Abstract

Cellular communication network (CCN) proteins are key matricellular regulators of cartilage development, yet their species‐specific roles and network‐level context remain unclear. This study integrated bulk RNA sequencing from chicken and mouse embryonic limb bud micromass cultures and human mesenchymal stem cell chondrogenesis with co‐expression, protein–protein interaction, and ortholog analyses to construct CCN‐centered regulatory networks across models. CCN1 and CCN2 emerged as dominant, conserved hubs enriched in collagen‐containing extracellular matrix, cartilage development, and growth factor signaling modules, whereas CCN3–CCN6 showed lower context‐dependent expression and connectivity. Functional and ortholog analyses revealed moderate pathway conservation, with high conservation of IGF, EGFR, and HIF‐1 signaling, but reduced overlap in hypoxia and mechanosensing/Hippo categories, indicating species‐specific tuning of environmental sensing. A focused ortholog screen identified multifunctional conserved hubs, including *COL2A1*, *TGFBR1*, *SMAD3*, *RUNX2*, *HIF1A*, *IGF1*, *SPP1*, and *CD44*. Single‐cell RNA‐seq meta‐analysis of human iPSC‐derived chondrogenesis and embryonic limb datasets showed CCN1/2 expression and homologous network activity peaking in mesenchymal and early chondrocyte populations, consistent with model‐dependent persistence into hypertrophic and ossification stages in vivo. Overall, this work defines a conserved CCN1/2‐centered axis integrating extracellular matrix formation with growth factor and mechanical cues, providing a framework for model selection and CCN‐targeted cartilage regeneration strategies.

## Graphical abstract

Cross‐species network analysis identifies CCN1/2 as conserved hubs linking extracellular matrix assembly, growth factor signaling, and mechanosensitive pathways during chondrogenesis, guiding model selection and informing CCN‐targeted cartilage regeneration strategies.

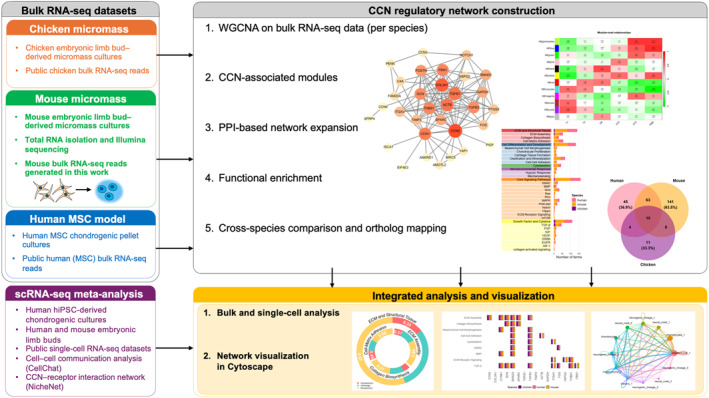

## 1 INTRODUCTION

Musculoskeletal disorders such as osteoarthritis (OA) cause chronic pain, loss of joint function, and reduced mobility, posing a major global health burden.^1^ The limited regenerative capacity of articular hyaline cartilage highlights the critical need for innovative approaches to cartilage repair and regeneration.^2^, ^3^ Understanding the molecular mechanisms underlying chondrogenesis is crucial for developing effective therapeutic strategies for cartilage disorders.^4^, ^5^

Chondrogenesis is a complex process in which chondroprogenitor cells differentiate into chondrocytes that produce and maintain the cartilage extracellular matrix (ECM).^5^, ^6^ This process involves a complex interplay of signaling pathways, transcription factors, and ECM components.^7^, ^8^, ^9^ Among these regulators, the cellular communication network (CCN) family of matricellular proteins has emerged as a key modulator of cartilage development and homeostasis.^10^, ^11^

The CCN family consists of six members: CCN1 (CYR61), CCN2 (CTGF), CCN3 (NOV), CCN4 (WISP1), CCN5 (WISP2), and CCN6 (WISP3).^12^, ^13^, ^14^ These proteins are characterized by a modular structure consisting of four conserved domains: insulin‐like growth factor binding protein (IGFBP), von Willebrand factor type C repeat (VWC), thrombospondin type 1 repeat (TSP1), and a cysteine‐knot‐containing (CT) domain.^15^ This unique structure allows CCN proteins to interact with a wide range of molecules, including growth factors, ECM components, and cell surface receptors, thereby modulating various cellular processes.^16^

CCN proteins play crucial roles in diverse biological processes, including cell adhesion, migration, proliferation, differentiation, and survival.^17^, ^18^ In the context of chondrogenesis and cartilage homeostasis, CCN proteins have been shown to regulate key aspects of chondrocyte biology and ECM production.^10^, ^11^ Despite structural conservation, CCN family members display species‐dependent expression and functions during chondrogenesis, highlighting the need for comparative studies to elucidate their roles in different model systems.^19^ For instance, both CCN1 and CCN2 contribute to cartilage formation but exert distinct effects depending on species and developmental context.^20^, ^21^, ^22^, ^23^, ^24^, ^25^, ^26^

The roles of other CCN family members in chondrogenesis are less well‐characterized and also exhibit variable chondrogenic regulation across human, murine, and avian systems.^27^, ^28^, ^29^, ^30^, ^31^, ^32^ Given the complex and species‐dependent functions of CCN proteins in chondrogenesis, there is a clear need for comparative studies to elucidate the conservation and divergence of CCN‐mediated regulatory networks across different, widely used model systems. Such a study is crucial for assessing the applicability of various in vitro chondrogenic models to human cartilage biology and for developing targeted therapeutic strategies for cartilage repair and regeneration.

In vitro models of chondrogenesis, such as chicken and mouse embryonic limb bud micromass cultures and human mesenchymal stem cell (MSC)‐derived chondrocytes, have been powerful tools for studying the molecular mechanisms underlying cartilage formation and homeostasis.^12^, ^33^, ^34^, ^35^, ^36^, ^37^ Each model recapitulates specific aspects of chondrogenesis: The avian system is well‐suited for studying early differentiation events, the mouse model allows for genetic manipulation, and human MSC cultures provide direct translational relevance.^38^, ^39^, ^40^, ^41^, ^42^, ^43^

Although each of these models has contributed significantly to our understanding of chondrogenesis, there is a need for integrative cross‐species analyses that quantify how far their underlying regulatory programs are conserved and how faithfully they capture human CCN biology. In the case of CCN proteins, whose matricellular functions are highly context‐ and species‐dependent, it is particularly important to move beyond single‐gene descriptions and to define their position within broader co‐expression and signaling networks. In this study, bulk RNA sequencing of chicken and mouse embryonic limb bud micromass cultures and human MSC‐derived chondrocytes was combined with co‐expression analysis, protein–protein interaction (PPI) networks, functional enrichment, and ortholog mapping to construct CCN‐centered regulatory networks in each species. In addition, single‐cell RNA‐sequencing (RNA‐seq) meta‐analysis of hiPSC‐derived human chondrogenesis was used to resolve the cell‐state specificity, temporal dynamics, and ligand–receptor interactions of the human CCN regulatory module. Together, this comparative framework identifies a conserved CCN1/2‐centered axis that links ECM assembly with growth factor and mechanotransduction pathways, delineates species‐ and model‐specific extensions in hypoxia and mechanosensing networks, and provides an evidence‐based assessment of the translational strengths and limitations of each in vitro chondrogenic system for human cartilage research.

## 2 MATERIALS AND METHODS

### 2.1 Study design

Bulk RNA‐seq and single‐cell RNA‐seq datasets were analyzed in this study to characterize CCN‐centered regulatory programmes in chondrogenic systems from three species. For the chicken micromass model, previously published bulk RNA‐seq datasets were used (BioProject IDs: PRJNA817177; PRJNA938813).^8^ Human MSC chondrogenic differentiation data were obtained from th egene expression omnibus database (GSE109503).^35^ Primary murine micromass cultures were established from limb bud‐derived mesenchymal progenitors (LMPs), and bulk RNA‐seq was performed to profile their chondrogenic gene expression signatures at key time points of differentiation (days 1, 3, 6, and 10) using Illumina sequencing.

**FIGURE 1:**
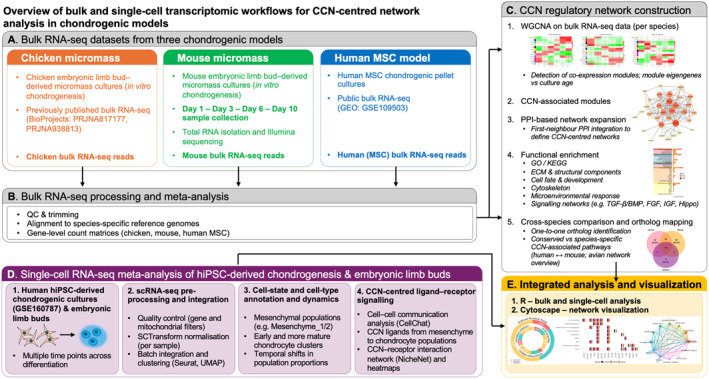
Overview of the experimental and bioinformatic workflow integrating bulk RNA‐seq from chicken, mouse, and human MSC chondrogenic models with CCN‐centered network analyses and single‐cell RNA‐seq meta‐analysis of hiPSC‐derived chondrogenesis.

Using bulk RNA‐seq data from the three species, CCN protein regulatory networks were constructed by integrating weighted gene co‐expression network analysis (WGCNA) with first‐neighbor PPI analysis, followed by functional enrichment analysis, cross‐species comparison of functional categories, and ortholog mapping of conserved and species‐specific components. In parallel, single‐cell RNA‐seq data from hiPSC‐derived human chondrogenesis (GSE160787), as well as human and mouse embryonic limb single‐cell RNA‐seq data obtained from previously published quality‐controlled and annotated public datasets were analyzed to resolve CCN family expression, CCN network activity, and CCN‐centered ligand–receptor signaling at cell‐state and cell‐type resolution across the differentiation time course. All computational analyses and visualizations were performed using R (version 4.5.1) and Cytoscape (version 3.10.4), and the integrated experimental and bioinformatic workflow is summarized in Figure 1.

### 2.2 Mouse micromass cultures

The mouse micromass culture system was used to study in vitro chondrogenesis, following the protocol established by Vágó et al.^37^ All animal studies were approved by the Wonkwang University Animal Care and Use Committee (#WKU18‐23, WKU19‐09, WKU20‐61) and were in compliance with the institutional guidelines. Limb buds were isolated from wild‐type E11.5‐E12.5 mouse embryos (C57BL/6N) and enzymatically digested with 1% trypsin‐EDTA (Merck, USA) for 30 min at 37°C to obtain a single‐cell suspension of LMP cells. The resulting suspension was filtered through a 40‐μm cell strainer (Merck) and cell numbers were determined using an automated cell counter (Logos Biosystems, South Korea).

High‐density micromass cultures were prepared by spotting 100 μL droplets containing 10^6^ cells (10^7^ cells/mL) into 6‐well tissue culture plates. After a 2‐h attachment period in a humidified incubator (37°C, 5% CO_2_), 2 mL of culture medium was gently added. The culture medium consisted of high‐glucose DMEM (Capricorn Scientific, Germany) supplemented with 10% fetal bovine serum (Thermo Fisher Scientific, Waltham, MA, United States) and 1% penicillin‐streptomycin (Merck). Medium was changed every other day, and cultures were maintained for up to 10 days to allow chondrogenic differentiation and cartilage nodule formation.

Morphological changes and ECM production were monitored throughout the culture period using Alcian blue staining. For staining, cultures were fixed in Kahle's fixative and incubated overnight with 1% Alcian blue in 0.1 N HCl. Excess dye was removed by washing with distilled water, the stained cultures were air‐dried, and photomicrographs were taken using a Leica light microscope (see Figure S1).

### 2.3 RNA extraction and bulk RNA‐seq

On culture days 1, 3, 6, and 10, TRI Reagent (Applied Biosystems, Foster City, CA, USA) was added directly to micromass cultures, and the samples were stored at −80°C until processing. For total RNA isolation, lysates were mixed with 20% chloroform, followed by centrifugation at 10,000 × *g* for 20 min at 4°C. The aqueous phase was precipitated with 500 μL of RNase‐free isopropanol and incubated for 1 h at −20°C. The resulting RNA pellets were dissolved in RNase‐free water (Promega, USA) and stored at −80°C.

Bulk mRNA sequencing was performed as described previously.^8^, ^44^ RNA quality was assessed using the Eukaryotic Total RNA Nano Kit on an Agilent BioAnalyzer (Santa Clara, CA, USA) according to the manufacturer's instructions. Only samples with RNA integrity number (RIN) values greater than 7 were used for library preparation. Sequencing libraries were prepared from total RNA using the Ultra II RNA sample prep kit (New England BioLabs, USA). Sequencing was carried out on an Illumina NextSeq 500 platform using single‐end 75 cycle runs, yielding an average of approximately 20 million raw reads per sample. HISAT2 aligner was used to map the raw counts to the GRCm38 (mm10) mouse reference genome. The entire dataset has been uploaded to the NCBI SRA database, under the following BioProject ID: PRJNA1421121 (http://www.ncbi.nlm.nih.gov/bioproject/1421121).

### 2.4 Bioinformatics

Mapped raw count data from mouse were normalized using the *DESeq2* package (version 1.48.2). Normalized data for the human (GSE109503) and the chicken model (PRJNA817177; PRJNA938813) were obtained from published resources. First, principal component analysis (PCA) was performed separately for the cell populations of each species using the *prcomp* function to evaluate the temporal transcriptional dynamics within their respective in vitro chondrogenic differentiation models. CCN family genes were then screened, and their expression patterns were visualized using heatmaps (generated with the *pheatmap* package, version 1.0.13) and line plots (visualized with *ggplot2*, version 4.0.1).

To construct *CCN protein regulatory networks* for each of the three species, the signed hybrid network algorithm of WGCNA (via the *WGCNA* package, version 1.73) was first applied to identify co‐expressed gene modules. This approach clustered RNA‐seq data into distinct modules represented by module eigengenes (MEs) based on similar expression patterns. We specifically focused on the MEs containing CCN family genes. Bootstrap resampling (10 iterations with 80% of samples) was used to evaluate WGCNA sensitivity. A single 10% leave‐out analysis was additionally performed to assess the stability of MEs and to quantify the retention rate of genes in the CCN‐centered networks. Bootstrap resampling produced Jaccard indices of 0.417 for human, 0.481 for mouse, and 0.464 for chicken, indicating moderate global network stability. In the 10% leave‐out analysis, genes within the key turquoise module remained predominantly assigned to their original module eigengene. Furthermore, the CCN‐centered networks showed substantial preservation, with retention rates of 64.5% (human), 72.2% (mouse), and 74.2% (chicken).

All genes within each of these CCN‐associated MEs were used to separately construct PPI networks via the *STRING* database. We set the STRING interaction confidence threshold to 0.4, which corresponds to medium–high confidence and is widely used for exploratory PPI network reconstruction,^45^ because our aim was to capture a sufficiently dense first‐neighbor landscape around CCN genes while retaining experimentally and literature‐supported interactions. At higher cut‐offs (0.7–0.9), many context‐relevant but less extensively studied interactions, especially in cartilage and for non‐canonical regulators, are lost and the networks become too sparse for meaningful module‐level comparison across species, whereas a 0.4 threshold preserves the overall topology and CCN1/CCN2 hub status while still restricting the analysis to interactions supported by multiple evidence channels in STRING.

The individual PPI networks were then sequentially imported into *Cytoscape* for independent analysis. For each ME, we identified the proximal associated proteins of its corresponding CCN family member(s). These proximal proteins were defined as those exhibiting non‐directional interactions with the CCN member(s) within that specific ME at the protein level, while also sharing highly correlated expression patterns at the transcriptomic level. Finally, the interconnected first‐neighbor subsets from all analyzed MEs were merged to form the comprehensive CCN protein regulatory network in chondrogenic cell cultures.

To functionally characterize this network, gene ontology (GO) and KEGG pathway enrichment analyses were performed using the *clusterProfiler* package (version 4.16.0). Significantly enriched GO terms and KEGG pathways (adjusted *p*‐value <0.05, FDR‐corrected) related to chondrogenic processes were filtered and summarized into five principal functional categories: (1) ECM and structural tissue constituent; (2) cell fate, differentiation and development; (3) cytoskeleton; (4) microenvironmental response; and (5) signaling networks. Cross‐species functional comparative analysis was subsequently conducted.

Orthologous gene annotation was obtained using the R package *biomaRt* (version 2.64.0) to retrieve reciprocal best‐hit gene pairs across species. To ensure strict orthologous relationships, only genes showing one‐to‐one mapping in bidirectional queries were retained and considered as conserved orthologs across species. Cross‐species PPI networks were similarly constructed via the *STRING* database (confidence set to 0.4), integrated and visualized using *Cytoscape*.

### 2.5 Single‐cell RNA‐seq data processing and CCN‐centered analyses

Single‐cell RNA sequencing data from human induced pluripotent stem cell (hiPSC)‐derived chondrogenic cultures (GSE160787) were obtained from a publicly available dataset. Cells from all time points of the differentiation protocol were included. We followed the processing workflow and filtering criteria published in the original study.^9^ Low‐quality cells (with less than 200 or more than 7000 detected genes or if their mitochondrial gene content was more than 5%) were removed, and genes that were detected in less than three cells were excluded prior to downstream analysis. Cell cycle phase scores (S phase and G2/M phase) were first computed using Seurat's *CellCycleScoring* function. These scores were subsequently regressed out during *SCTransform* normalization, where each sample was independently normalized and variance‐stabilized in Seurat v5 while controlling for mitochondrial gene content, S.Score, and G2M.Score. Samples were then integrated to mitigate batch effects. Briefly, 3000 highly variable features were selected, and integration anchors were identified using SCT‐corrected data. For downstream analysis, cells were clustered using an unsupervised graph‐based approach and visualized in two dimensions with UMAP, based on the first 40 principal components and a clustering resolution of 0.6. Cell clusters were annotated based on the original study^9^ and using the expression of canonical mesenchymal and chondrocyte markers.

To evaluate the differentiation trajectories of each cell population over time, we used time points as grouping variables and calculated the proportion of cells in each population relative to the total number of cells per group. Visualization was performed using bar plots generated with the *ggplot2* package. To assess the expression of CCN family genes at single‐cell resolution, the *FeaturePlot* function was used to visualize the expression distribution of CCN1–CCN6 on UMAP embeddings. Given that the *CCN protein regulatory network* is closely associated with the functions of CCN family proteins, we constructed this network based on human bulk RNA‐seq data. Its activity level in each cell was subsequently calculated using the *AUCell* package and visualized.

Intercellular communication strength among the overall cell populations was analyzed with the *CellChat* package. We paid particular attention to CCN family genes acting as ligands in the signaling communication between mesenchymal cells and chondrocytes. Therefore, using the *nichenetr* package, Mesenchyme_1 and Mesenchyme_2 were defined as sender populations, whereas chondrocytes_1 and chondrocytes_2 were specified as receiver populations, thereby constructing a CCN–receptor interaction network. Based on this network, a heatmap was used to display the expression patterns of ligands and receptors.

Human embryonic limb single‐cell RNA‐seq data were obtained from a previously published quality‐controlled and annotated public dataset.^46^ Mouse embryonic limb single‐cell data were retrieved from GSE142425^47^ and processed using the same normalization and quality‐control pipeline. Cells expressing fewer than 200 genes or more than 6000 genes, as well as cells with mitochondrial gene content exceeding 10%, were excluded. Dimensionality reduction was performed based on the first 30 principal components, followed by clustering with a resolution of 0.5. In addition, activity levels in the human embryonic limb dataset were evaluated using the *UCell* package.

## 3 RESULTS

### 3.1 Transcriptomic profiling of in vitro chondrogenic models

**FIGURE 2:**
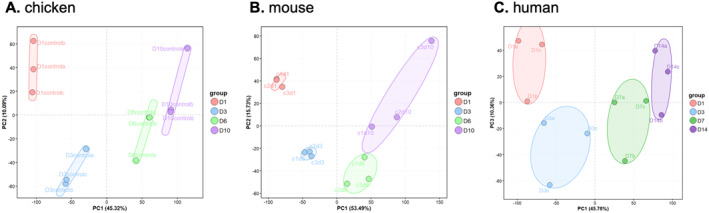
Comparative transcriptomic analysis of three in vitro chondrogenic differentiation models: (A) chicken embryonic limb bud‐derived micromass model; (B) mouse embryonic limb bud‐derived micromass model; and (C) human mesenchymal stem cell model. Principal component analysis plots from normalized bulk RNA‐seq data demonstrate unsupervised clustering by culture age (maturation), with chicken and mouse models shown in PC1–PC2 space and the human model shown in PC1–PC3 space to highlight the shared differentiation‐associated trajectory across species. Each point represents a biological replicate (*N* = 3).

Comprehensive transcriptomic profiling was performed across three in vitro chondrogenic differentiation models. PCA revealed robust and reproducible transcriptomic shifts accompanying chondrogenic differentiation in all systems, which corroborate gradually increasing ECM production in the mouse model (Figure S1). Variability between time points, representing culture maturation, exceeded inter‐replicate variation (Figure 2). Importantly, when the three datasets were examined in a common PCA framework, all models displayed broadly comparable trends in overall transcriptomic organization, aligning along similar trajectories in higher‐order components (notably PC2 and PC3), supporting the use of these systems for integrated cross‐species analyses.

Consistent with previous studies,^8^, ^35^ unsupervised clustering in the murine micromass model demonstrated continuous maturation trajectories along the PC1 axis from the undifferentiated (day 0) to mature (day 10) states. Highly similar transcriptional trajectory patterns were observed between murine and avian systems, with parallel distributions across both PC1 and PC2 dimensions, suggesting conserved transcriptional regulation in these embryonic limb bud‐derived systems (Figure 2A,B). The human MSC model system progressed along a comparable primary differentiation axis and projected in parallel to the embryonic trajectories in additional components (Figure 2C), supporting the use of this model in integrated cross‐species analyses of CCN‐centered regulatory networks.

### 3.2 Expression dynamics of CCN family genes across chondrogenic models

**FIGURE 3:**
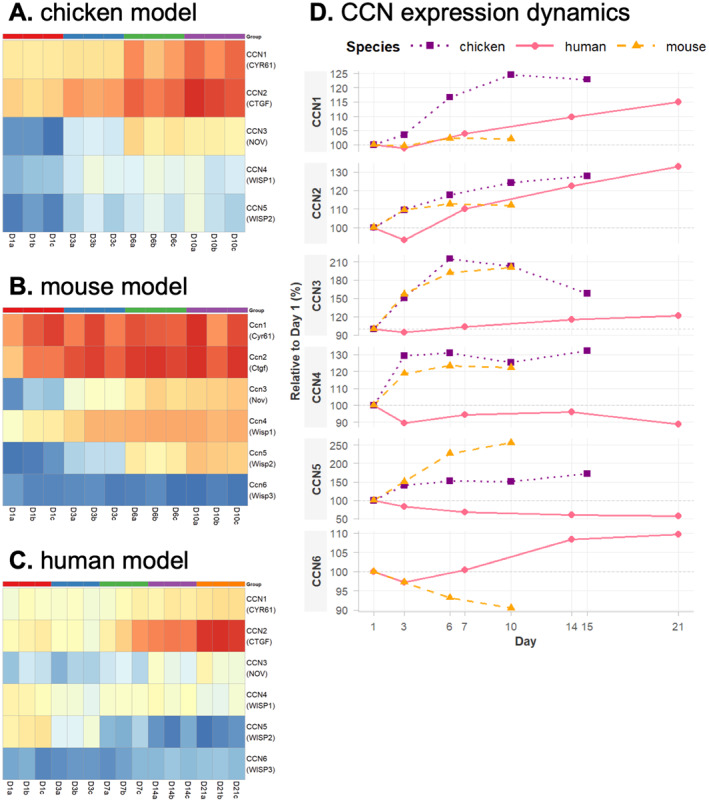
CCN gene expression dynamics across chondrogenic models. (A–C) Heatmaps of CCN family gene expression during in vitro chondrogenesis in (A) chicken embryonic limb bud‐derived micromass, (B) mouse embryonic limb bud‐derived micromass, and (C) human mesenchymal stem cell cultures. Each heatmap shows normalized transcript counts across differentiation time points (days; chicken: D1–D10; mouse: D1–D10; human: D1–D21; *N* = 3 biological replicates per time point). (D) Line plots summarizing relative temporal expression changes of individual CCN genes across species, expressed as percentage of day 1 levels, highlighting conserved CCN1/CCN2 upregulation and model‐specific regulation of CCN3–CCN6. CNN, cellular communication network.

Transcriptional profiling revealed distinct and model‐dependent expression patterns among CCN family members during chondrogenic differentiation. In the avian micromass model, transcripts corresponding to *CCN1–5* were detected, whereas *CCN6* (*WISP3*) was absent, consistent with the lack of an annotated ortholog in the chicken genome. *CCN1* and *CCN2* exhibited the highest expression levels, both showing progressive upregulation during late stages of differentiation (days 6–10). *CCN3* and *CCN5* displayed modest induction at lower transcript abundances, whereas *CCN4* showed variable non‐directional changes throughout the chondrogenic timeline (Figure 3A). In the mouse micromass model, all six CCN orthologs were expressed, reflecting a similar dominance of *Ccn1* and *Ccn2*, alongside induction of *Ccn3–5* at later stages of chondrogenesis (Figure 3B). The human MSC‐based model demonstrated conserved *CCN2* upregulation during terminal differentiation (days 14–21), with moderate increases in *CCN1* and *CCN3* expression. In contrast to the embryonic models, *CCN5* was progressively downregulated during culture maturation, and *CCN4* again exhibited no consistent temporal trend (Figure 3C). *CCN6* expression remained low and relatively unchanged. When CCN expression was compared across species in a unified relative scale, CCN1 and CCN2 showed conserved upregulation during chondrogenic progression, whereas CCN3–CCN6 displayed divergent model‐dependent dynamics (Figure 3D).

### 3.3 Gene co‐expression networks in chondrogenic systems

To place CCN genes into their broader regulatory context, WGCNA was performed using culture age as the trait. This approach revealed distinct clusters of genes highly correlated with individual CCN members in all three models, whose module eigengenes (MEs) were significantly correlated with chondrogenic maturation and contained one or more CCN family members, indicating coordinated regulation with larger gene sets rather than isolated expression changes. In the avian model, the five expressed CCN genes (*CCN1–CCN5*) were distributed among three modules showing significant correlation with the differentiation trajectory (Figure S2A). Specifically, *CCN1* and *CCN2* localized to the turquoise ME, *CCN3* and *CCN4* to the yellow ME, and *CCN5* to the green–yellow ME. In the murine chondrogenic model, all six CCN orthologs were distributed across 2 MEs displaying strong correlation with the trait. *Ccn1* was assigned to the magenta ME, while all other CCN genes were localized to the turquoise ME (Figure S2B). In the human MSC model, all six CCN genes were distributed across three significantly correlated MEs. *CCN1*, *CCN2*, *CCN3* and *CCN5* were assigned to the turquoise ME, *CCN4* to the black ME, and *CCN6* to the purple ME (Figure S2C). These CCN‐containing modules formed the basis for subsequent PPI network reconstruction and functional enrichment analyses.

### 3.4 CCN protein regulatory networks and core hub genes

**FIGURE 4:**
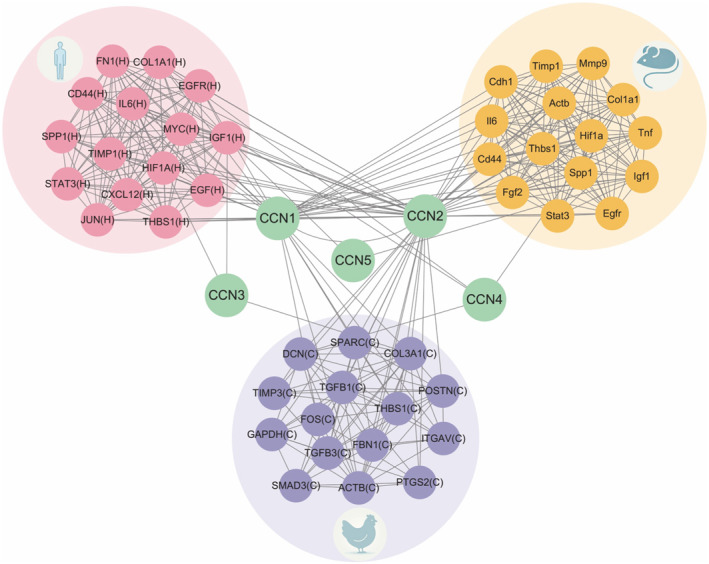
Core hub genes in CCN‐centered regulatory networks. Top 15 most connected proteins within CCN regulatory networks derived from weighted gene co‐expression network analysis modules and first‐neighbor protein–protein interaction analysis for human hMSCs (top left, red), mouse micromass (top right, orange), and chicken micromass cultures (bottom, purple), ranked by degree centrality. CCN1 and CCN2 rank among the highest‐degree nodes in all species. Conserved mammalian hubs include collagens, matricellular proteins, growth factors, and signaling effectors. Complete network data are provided in Figure S3. CNN, cellular communication network.

To systematically identify and characterize the functional landscape of *CCN‐centered regulatory networks*, we constructed CCN protein regulatory networks for all three species by combining WGCNA module assignment with first‐neighbor PPI analysis from the STRING database. These networks encompass 122 genes in humans, 222 genes in mouse, and 33 genes in chicken (Figure S3; gene/protein lists are provided in Table S1), and represent the interconnected set of proteins exhibiting direct physical interactions with CCN family members while sharing highly correlated expression patterns during chondrogenic differentiation. Degree analysis of these networks revealed that CCN1 and CCN2 consistently emerged as the most highly connected hub nodes across all three species, with substantially higher connectivity than other CCN family members (Figure 4). This finding reinforces their dominant functional roles identified in the bulk expression analysis and suggests that CCN1/2‐mediated signaling represents a central regulatory axis within the broader chondrogenic transcriptional landscape (Figure S3).

Notably, there were substantial overlaps in the identity of high‐degree interactants between human and mouse networks, with shared hub proteins including collagens (COL1A1), matricellular proteins (THBS1, SPP1), growth factor regulators and cytokine regulators (IGF1, IL6, EGFR), signaling mediators (STAT3), cell adhesion molecules (CD44), ECM remodeling regulators (TIMP1), and hypoxia‐responsive factors (HIF1A) (Figure 4). This high degree of network conservation at the level of individual interacting partners suggests that CCN‐mediated regulatory mechanisms are substantially preserved across mammalian lineages. By contrast, the chicken network comprised considerably fewer nodes and lacked several of these conserved interactants, likely reflecting incomplete gene annotation in the chicken genome assembly and the deeper phylogenetic distance between birds and mammals.

### 3.5 Functional classification and enrichment of CCN networks across species

**FIGURE 5:**
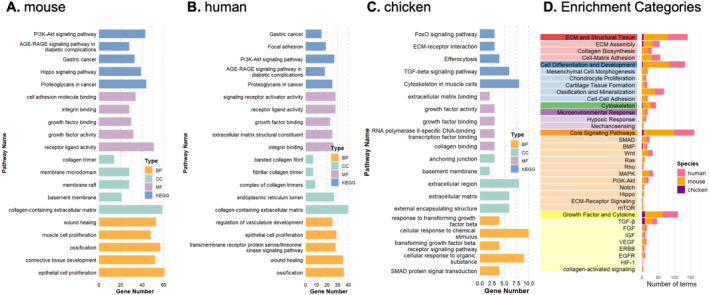
Top enriched functional terms within cellular communication network regulatory networks. (A–C) Bar charts showing the 5 most significantly enriched gene ontology/KEGG terms for mouse, human and chicken networks, ordered by enrichment *p*‐value (FDR‐corrected) and colored by functional category. (D) Hierarchical classification scheme organizing enriched terms into 5 major categories (ECM and structural tissue constituent, cell fate/differentiation/development, cytoskeleton, microenvironmental response, signaling networks) with subcategories and pathway‐specific subclasses. Detailed counts of enriched terms and genes within each category are provided in Table S2.

To characterize the biological functions of the CCN protein regulatory networks, we performed GO and KEGG pathway enrichment analyses and organized the resulting terms into five main functional categories: (1) ECM and structural tissue constituent; (2) cell fate, differentiation, and development; (3) cytoskeleton; (4) microenvironmental response; and (5) signaling networks (further subdivided into core pathways such as PI3K–Akt, MAPK, Hippo, and Rho signaling, and growth factor/cytokine signaling modules including TGF‐β, FGF, IGF signaling, and additional related pathways) (Figure 5). Detailed counts of enriched terms and genes within each category are provided in Table S2. In human and mouse, the top enriched terms within CCN regulatory networks were remarkably similar and dominated by ECM organization, collagen biosynthesis, cartilage tissue formation, growth factors, and ossification pathways (Figure 5A,B). This parallel enrichment profile underscores the conserved role of CCN‐centered signaling in orchestrating cartilage matrix assembly and mineralization. The murine network showed particularly strong representation of mechanosensitive Hippo and PI3K–Akt signaling together with integrin, adhesion, and collagen‐containing ECM terms, whereas the human MSC network was enriched for receptor‐mediated signaling, focal adhesion, and vascular/ECM‐related pathways, consistent with a culture context where growth factor and adhesion cues guide chondrogenic differentiation. The avian network exhibited sparse enrichment information, consistent with its reduced gene complement (Figure 5C). The few enriched terms identified in the chicken network were largely restricted to TGF‐β signaling, SMAD protein signal transduction, and collagen/ECM categories, limiting detailed functional comparison with mammals (Figure 5D). For this reason, subsequent conservation and pathway‐level analyses focused on humans and mice, with chickens retained mainly for genome‐level network summaries (Table S3).

### 3.6 Conservation of CCN regulatory networks across mammalian species

To assess how CCN‐associated regulatory mechanisms are shared between humans and mice, we performed cross‐species ortholog mapping within each functional category and quantified conservation using three complementary metrics: (1) A human‐centered conservation index, (2) a mouse‐centered conservation index, and (3) an overall Jaccard index that captures the fraction of genes shared between the two species (Table S4). Overall conservation was moderate (Jaccard values typically ∼0.25–0.30), indicating that roughly one quarter to one third of CCN‐associated genes in a given pathway are shared, with human‐centered conservation indices generally higher than the mouse‐centered ones (Table S4), suggesting that a substantial proportion of human CCN‐network components have identifiable mouse orthologs, whereas the murine networks contain a larger fraction of mouse‐specific extensions.

**FIGURE 6:**
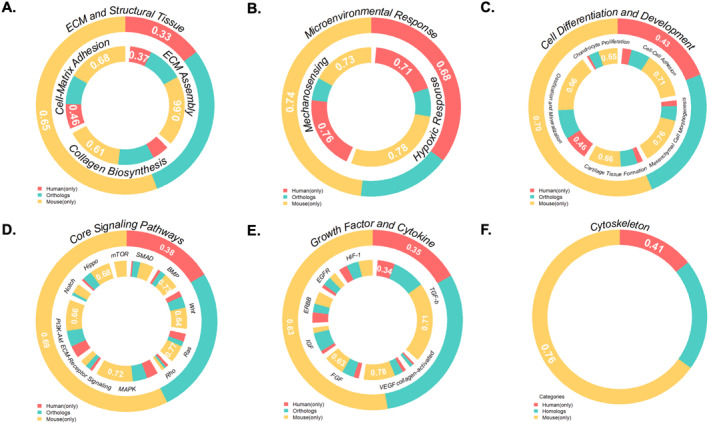
Ortholog conservation landscape in human and mouse CCN networks. Nested donut charts summarize, for each functional category and selected subclasses, the proportions of CCN‐associated genes classified as shared orthologous pairs present in both human and mouse (green), human‐only genes lacking mouse orthologs (red), or mouse‐only genes lacking human orthologs (yellow). Panels A–F illustrate the heterogeneous conservation patterns across broad categories, with relatively high shared content in several ECM‐related, developmental, and growth factor/signaling modules (e.g., IGF, EGFR, HIF‐1, ECM–receptor, and collagen‐activated signaling) and markedly lower overlap in microenvironmental response and cytoskeleton pathways. The outer ring in each panel represents the overall category, whereas inner rings display selected subclasses together with their species‐specificity indices (human or mouse). Data for all functional categories and pathways are provided in Tables S4 and S5, enabling detailed comparison of conserved versus species‐specific components of human and mouse CCN regulatory networks. CNN, cellular communication network.

Growth factor and signaling pathways were among the most conserved modules: IGF, EGFR, SMAD and related “chondrocyte proliferation and early differentiation” and Hippo categories showed high human and mouse conservation ratios and the largest Jaccard overlaps, whereas microenvironmental response and cytoskeleton‐related categories were markedly less conserved, reflecting species‐specific tuning of hypoxia, mechanosensing, and cytoskeletal regulation. ECM‐related modules showed intermediate conservation, with substantial shared cores for ECM organization, collagen biosynthesis, and cell–matrix adhesion but a notable contribution from species‐restricted genes, particularly in the mouse network (Figure 6; Tables S4 and S5).

### 3.7 Pathway‐specific PPI networks and ortholog mapping

**FIGURE 7:**
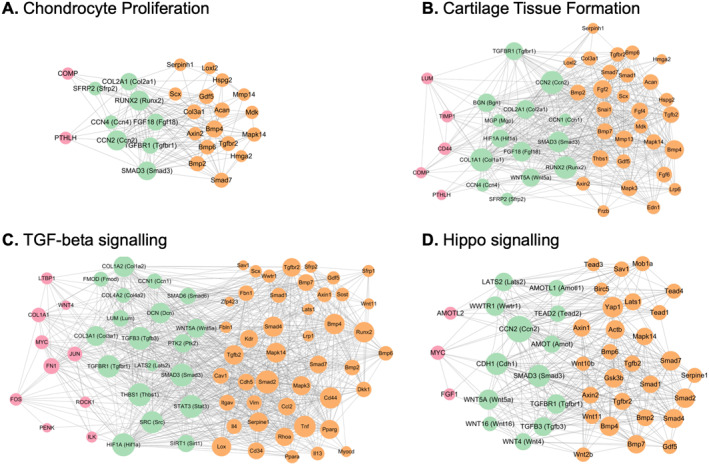
Pathway‐specific protein–protein interaction networks with ortholog mapping. (A–D) Network graphs displaying protein–protein interactions for representative pathways of biological interest: (A) chondrocyte proliferation; (B) cartilage tissue formation and patterning; (C) TGF‐β Signaling; and (D) Hippo/YAP mechanotransduction. Node color indicates ortholog status (green = both species, red = human‐only, yellow = mouse‐only). Figures S4–S8 show networks for additional pathways.

To provide mechanistic insight into how CCN proteins integrate with conserved and divergent regulatory pathways, we reconstructed PPI networks for selected functional pathways of high biological interest color‐coding nodes to distinguish orthologous gene pairs (green) from human‐only (red) and mouse‐only (yellow) genes. Representative pathways include “Chondrocyte proliferation,” “Cartilage tissue formation,” “TGF‐β signaling,” and “Hippo signaling” (Figure 7), whereas additional pathways are presented in Figure S4–S8. Within the “Chondrocyte proliferation” network, CCN2 and CCN4 form a central hub linking PTHLH and BMP/TGF‐β inputs to RUNX2, SMAD3, TGFBR1, FGF18, and core matrix genes such as COL2A1 and ACAN, with most canonical chondrogenic regulators present as conserved orthologs (Figure 7A). In the “Cartilage tissue formation” network, CCN1/2/4 connect collagen‐rich ECM components (COL1A1, COL2A1, COMP, and ACAN) and small leucine‐rich proteoglycans (BGN, LUM, MGP) to SMAD3, TGFBR1, and FGF18, indicating that CCN‐centered signaling coordinates matrix assembly with pro‐chondrogenic growth factor pathways in a broadly conserved mammalian module (Figure 7B). In the “TGF‐β pathway” network, a dense SMAD‐receptor core (SMADs, TGFBR1, and TGFB3) and associated ECM/signaling partners (e.g., CCN1, collagens, DCN, FMOD, LUM, THBS1, PTK2, SRC, and STAT3) is largely orthologous between humans and mice, whereas mouse‐specific ligands, co‐receptors, Hippo components, and inflammatory mediators extend this core toward mechanical and immune inputs (Figure 7C). CCN1 and its ECM partners lie at the interface between the conserved SMAD‐receptor module and integrin/FAK, cytoskeletal and Hippo‐YAP/TAZ nodes, consistent with a role for CCN proteins in tuning TGF‐β/BMP signal strength in a mechanosensitive environment.^48^ In the “Hippo pathway” network, CCN2 is embedded in a conserved LATS2‐TAZ module that integrates TGF‐β and Wnt signaling, whereas additional mouse‐specific Hippo kinases, Wnt ligands, and SMAD paralogs provide extra layers of control over proliferative and differentiation outputs (Figure 7D).

### 3.8 Multi‐functional hub genes: Key nodes in CCN regulatory networks

**FIGURE 8:**
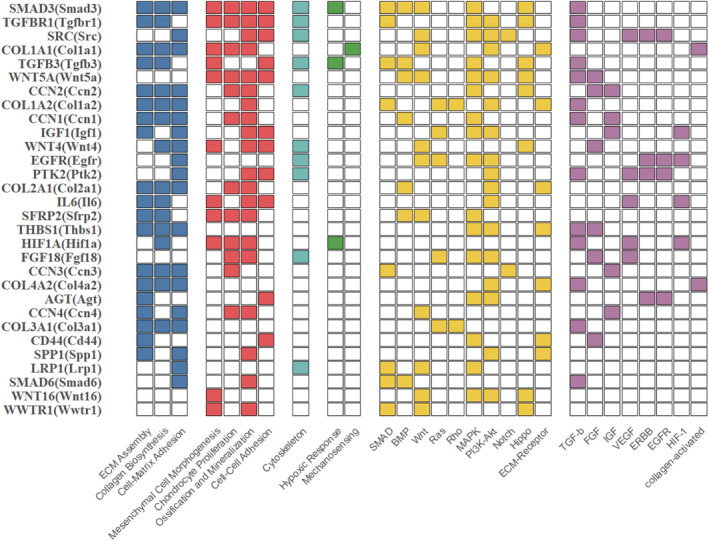
Multi‐functional hub genes integrating diverse chondrogenic pathways. Ranked bar chart displaying the top 30 orthologous genes present in the cellular communication network regulatory networks of both human and mouse that participate in multiple functional categories of interest (ECM, differentiation, signaling, microenvironmental response modules). The *y*‐axis shows gene symbol ordered by the number of distinct functional categories in which each gene appears; the *x*‐axis shows the functional categories. The chart shows which specific functional categories each gene participates in, enabling visual identification of shared regulatory mechanisms.

Network integration highlighted of a small cohort of orthologous genes that participate in multiple functional categories and are conserved between human and mouse, suggesting that they act as critical integration points for CCN‐centered signaling (Figure 8). Among these, core matrix constituents such as *COL2A1/Col2a1* and *COL1A1/Col1a1* appeared across ECM assembly, collagen organization, and cartilage development categories, while *TGFBR1/Tgfbr1* and *SMAD3/Smad3* repeatedly mapped to signaling and cell‐fate modules, consistent with a conserved TGF‐β axis controlling chondrogenic transcription. Additional hubs included *SRC/Src* in angiogenesis‐related pathways, *HIF1A/Hif1a* and *IGF1/Igf1* in hypoxic and growth factor signaling, and *SPP1/Spp1* together with *CD44/Cd44* in ECM assembly and adhesion categories. Collectively, these multi‐functional hubs link matrix production, growth factor responses and microenvironmental sensing, and provide plausible routes through which CCN networks coordinate structural, signaling, and environmental inputs during cartilage development and homeostasis.

### 3.9 Limited functional diversity in avian CCN networks

**FIGURE 9:**
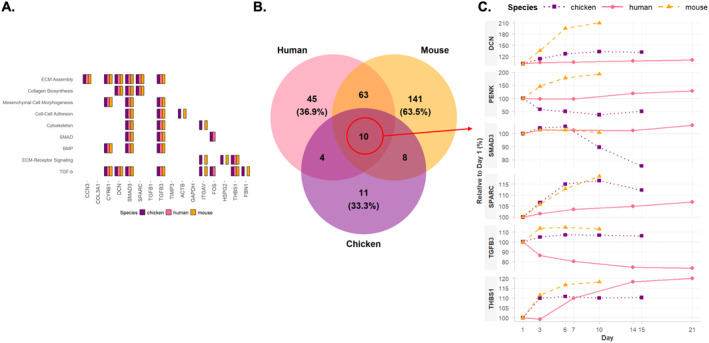
Limited functional coverage but conserved core dynamics of avian CCN regulatory networks. (A) Heatmap‐style overview of functional category assignments for CCN‐associated genes in human, mouse, and chicken networks, grouped into ECM assembly, collagen biosynthesis, mesenchymal cell morphogenesis, cell–cell adhesion, cytoskeleton, SMAD, BMP, ECM–receptor signaling, and TGF‐β modules. Each colored tile indicates the presence of a given gene in the corresponding functional term for the indicated species, illustrating the dense coverage of ECM and signaling functions in human and mouse compared with the sparse representation in chicken. (B) Venn diagram summarizing the overlap of CCN regulatory network, with only 10 orthologs shared across all three species (highlighted by red circle). (C) Line plots of relative transcript abundance (percent of day 1) for the six three‐way orthologs that participate in these functional categories (DCN, PENK, SMAD3, SPARC, TGFB3, THBS1) during chondrogenic differentiation in chicken, mouse, and human cultures. CNN, cellular communication network.

The avian CCN regulatory network showed markedly reduced functional coverage compared to mammalian networks. Only 16 genes from the chicken CCN regulatory network could be assigned to the functional categories of interest (ECM, differentiation, and signaling modules), in stark contrast to 72 human genes and 139 mouse genes (Figure 9). This 8–9‐fold reduction likely reflects incomplete *Gallus gallus* genome annotation, rather than a true biological depletion of functional complexity. To assess how this limitation translates into transcriptional dynamics, we compared six three‐way orthologs shared across all species (DCN, PENK, SMAD3, SPARC, TGFB3, and THBS1) (Figure 9). In human and mouse cultures, these genes generally displayed gradual sustained upregulation relative to day 1, consistent with progressive reinforcement of pericellular matrix organization (DCN, SPARC, and THBS1), canonical TGF‐β signal transduction (SMAD3), and CCN‐associated peptide regulation (PENK) during cartilage maturation. TGFB3 was particularly prominent in the embryonic micromass models. In chicken micromass cultures, however, regulation was weaker and often transient: DCN and TGFB3 exhibited only modest induction, PENK was downregulated, and SMAD3 and THBS1 peaked earlier and then declined, suggesting a narrower temporal window of CCN‐linked TGF‐β signaling and reinforcing that the avian model captures only a subset of the CCN‐centered programs active in mammalian chondrogenesis.

### 3.10 Single‐cell resolution of CCN‐mediated regulation during human chondrogenesis

**FIGURE 10:**
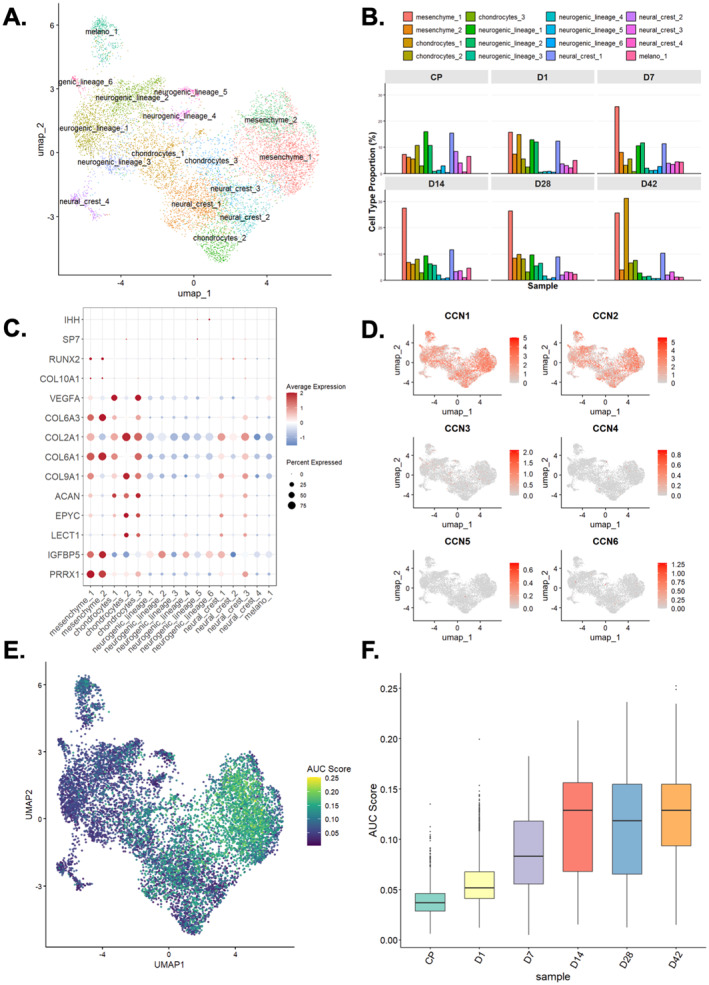
Single‐cell characterization of CCN family expression and CCN‐centered signaling during hiPSC chondrogenesis. (A) UMAP embedding of single‐cell RNA‐seq data from human hiPSC‐derived chondrogenic cultures (GSE160787), showing 10,008 cells clustered into 16 subpopulations, which were grouped into five major cell types: mesenchyme, chondrocytes, neural crest, neurogenic lineage, and melanocytes. (B) Bar plots showing the proportions of each subpopulation at individual time points (CP, D1, D7, D14, D28, D42), illustrating a marked expansion of mesenchyme_1 at D7 followed by an increase in chondrocytes_1 at D42. (C) Dot plot summarizing average expression and detection frequency of key mesenchymal, chondrogenic, and hypertrophic markers across all subpopulations, highlighting early chondrocyte‐like features in mesenchyme_1–2 and mature chondrocyte signatures in chondrocytes_1–3, with low expression of hypertrophic markers throughout. (D) UMAP feature plots of normalized expression for CCN family genes (CCN1–6), showing predominant CCN1 and CCN2 expression across the chondrogenic trajectory and much lower detection frequencies for CCN3–CCN6. (E) CCN protein regulatory network activity scored per cell using an AUCell‐based CCN gene set, demonstrating highest network activity in mesenchyme_1 and mesenchyme_2, and lower activity in the chondrocyte clusters. (F) Boxplots of CCN protein regulatory network scores across time points, indicating a pronounced increase in network activity from D7 with a peak at D14. CNN, cellular communication network.

To extend the bulk RNA‐seq findings to the cellular level, we analyzed single‐cell RNA sequencing (scRNA‐seq) data from human induced pluripotent stem cell (hiPSC)‐derived chondrogenic cultures (GSE160787). In total, 10,008 cells from chondrogenic progenitor (CP; *n* = 1932), day 1 (D1; *n* = 2199), D7 (*n* = 2148), D14 (*n* = 1242), D28 (*n* = 1328), and D42 (*n* = 1159) groups were clustered into 16 subpopulations, which were annotated as 5 major cell types: mesenchyme, chondrocytes, neurogenic lineage, neural crest, and melanocytes (Figure 10A). Analysis of subpopulation dynamics revealed a sharp expansion of the mesenchyme_1 population at D7, followed by a substantial increase in the proportion of the chondrocytes_1 population at D42 (Figure 10B). Marker analysis showed that mesenchyme_1–2 expressed classical mesenchymal markers and early chondrocyte‐like genes (such as *PRRX1*, *IGFBP5*, *COL6A3*, and *COL6A1*), whereas chondrocytes_1–3 were enriched for mature chondrocyte markers (such as *LECT1*, *EPYC*, and *ACAN*), and hypertrophic markers (*COL10A1*, *RUNX2*, *SP7*, and *IHH*) remained low throughout, indicating limited modeling of late hypertrophic or ossification stages (Figure 10C).

Across the differentiation trajectory, CCN1 and CCN2 were the predominant CCN transcripts at single‐cell level, with CCN3–CCN6 detected only in small fractions of cells (Figure 10D). CCN1/2 expression increased markedly at D7 and peaked around D14, coinciding with the emergence of chondrocyte characteristics, but the proportion of cells with high CCN1/2 expression declined by D42 (Figure S9). Scoring of the *CCN protein regulatory network* at single‐cell resolution revealed highest network activity in mesenchyme_1 and mesenchyme_2, and lower scores in mature chondrocytes (chondrocytes_1–3), suggesting that CCN‐driven signaling is most prominent in early mesenchymal and early chondrocyte states and tapers as cells mature (Figure 10E, Figure S10). Network activity at culture level began to rise markedly from D7 and reached its peak at D14, which closely coincides with the window of early chondrogenic commitment (Figure 10F).

**FIGURE 11:**
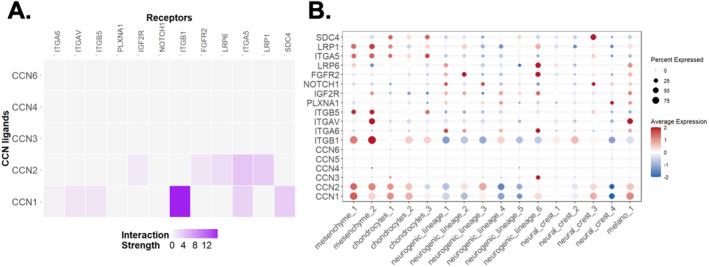
CCN‐centered ligand–receptor signaling between mesenchymal and chondrocyte populations in hiPSC chondrogenesis. (A) Heatmap of CCN ligand–receptor interaction strength inferred by a CCN‐focused *NicheNet* analysis, with mesenchyme_1/2 defined as sender populations and chondrocytes_1/2/3 as receivers. Interaction intensity is encoded by color, highlighting the CCN1 (CYR61)–ITGB1 axis as the strongest predicted CCN‐mediated signal, with additional contributions from receptors such as LRP1, SDC4, and ITGA5. (B) Dot plot showing average expression (color scale) and detection frequency (dot size) of CCN ligands (CCN1–CCN6) and candidate receptors across all single‐cell subpopulations. CNN, cellular communication network.

Given the mesenchymal origin of most CCN ligands, we next investigated the CCN‐mediated communication with mesenchyme_1/2 as sender and chondrocytes_1–3 as receiver populations. A CCN‐focused ligand–receptor analysis identified the *CCN1(CYR61)*‐*ITGB1* pathway as the strongest predicted interaction, with additional contributions from *LRP1*, *SDC4*, and *ITGA5* in the receiver cells (Figure 11A). Consistent with this, CCN1/2 were mainly expressed in mesenchymal and chondrocyte clusters, whereas integrin receptors such as ITGB1, ITGB5, and ITGAV were strongly enriched in mesenchyme_1/2 and broadly expressed in chondrocyte subclusters (Figure 11B).

### 3.11 Single‐cell resolution of CCN‐mediated regulation in tissue development in vivo

**FIGURE 12:**
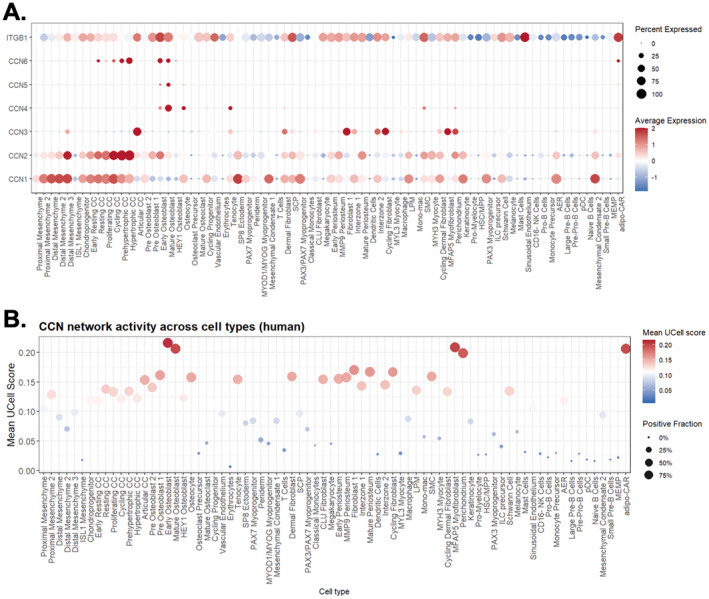
CCN expression and homologous CCN network activity in human embryonic limb development. (A) Dot plot showing average expression (color scale) and detection frequency (dot size) of CCN family ligands (CCN1–CCN6) and the key receptor ITGB1 across annotated cell types in human embryonic limb single‐cell RNA‐seq data. (B) CCN protein regulatory network activity (homologous human–mouse CCN network, defined from weighted gene co‐expression network analysis/protein–protein interaction analysis) scored per cell type using an AUCell‐based approach. CNN, cellular communication network.

To examine how the conserved CCN regulatory framework operates in vivo, we intersected the human and mouse CCN‐centered regulatory networks derived from WGCNA/PPI, to obtain a homologous CCN protein regulatory network containing only one‐to‐one orthologs, and scored its activity in single‐cell RNA‐seq datasets from human and mouse embryonic limbs. In human embryonic limb single‐cell data, CCN1/2 and the key receptor ITGB were highly expressed in mesenchymal and chondrocyte populations, whereas *CCN3*, *CCN4*, and *CCN6* showed more restricted patterns, enriched in articular chondrocytes, mature osteoblasts, and hypertrophic chondrocytes, respectively (Figure 12A). The homologous CCN protein regulatory showed high activity in proximal mesenchyme and chondroprogenitors, remained elevated in mature and hypertrophic chondrocytes, and peaked in ossification‐stage cell types, indicating sustained and progressively amplified engagement of the conserved CCN module during human skeletal maturation (Figure 12B).

**FIGURE 13:**
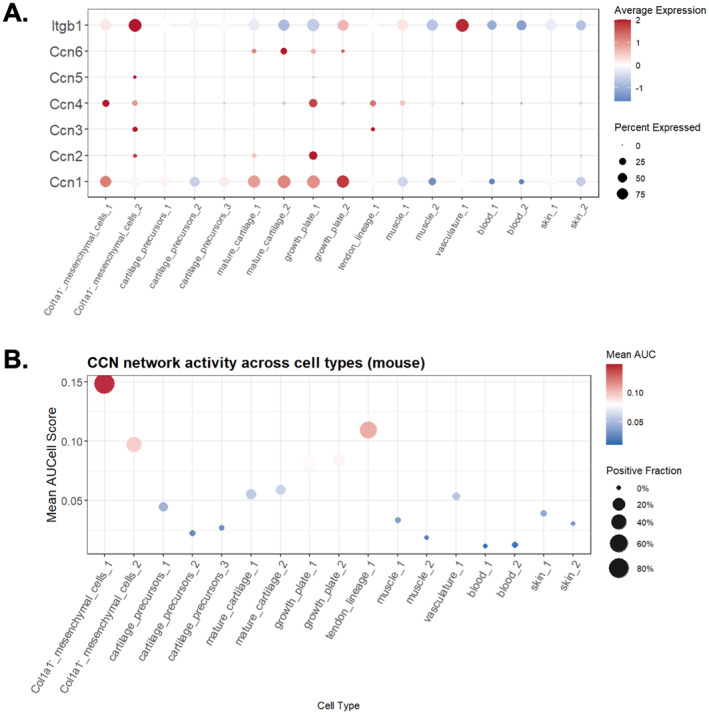
CCN expression and homologous CCN network activity in mouse embryonic limb development. (A) Dot plot showing average expression (color scale) and detection frequency (dot size) of Ccn1–Ccn6 and the key receptor Itgb1 across annotated mouse embryonic limb cell types. (B) CCN protein regulatory network activity (homologous human–mouse CCN network) scored per cell type using an AUCell‐based approach. CNN, cellular communication network.

In mouse embryonic limb single‐cell data, *Ccn1* and *Itgb1* similarly exhibited dominant expression in mesenchymal cells, mature and growth plate chondrocytes, whereas *Ccn2* wa strongly expressed only at the growth plate stage (Figure 13A). The activity of the homologous CCN protein regulatory network remained concentrated in Col1a1^+^ mesenchymal cells, displaying relatively low activity during cartilage formation, followed by a renewed increase in activity within the growth plate (Figure 13B).

## 4 DISCUSSION

The present study provides a systems‐level, cross‐species view of how CCN family members are embedded within chondrogenic regulatory programs, and introduces a network‐based framework for evaluating the translational relevance of commonly used in vitro and in vivo models. By integrating bulk RNA‐seq from avian and mammalian chondrogenic cultures, WGCNA‐based co‐expression analysis, PPI networks, ortholog mapping, and single‐cell data of hiPSC‐derived and embryonic limb chondrogenesis, the work moves beyond descriptive expression profiling and instead defines a conserved CCN1/2‐centered regulatory axis together with species‐ and context‐dependent extensions. The primary aim of the work is to establish and characterize this comparative network framework; mechanistic relationships inferred from it are therefore exploratory and will require targeted experimental validation.

### 4.1 CCN1/2 as core hubs in conserved chondrogenic programs

Across all bulk datasets, CCN1 and CCN2 were the most abundant and consistently upregulated CCN transcripts during chondrogenic maturation, whereas CCN3–CCN6 showed lower, more variable, or model‐specific expression. The observation that CCN1 and CCN2 dominate expression profiles across all three bulk models, and at single‐cell level in early hiPSC‐derived chondrocytes, reinforces their status as core regulators of cartilage development described in genetic and functional studies.^26^, ^49^, ^50^ Network reconstruction demonstrated that CCN1 and CCN2 occupy the highest‐degree positions in CCN protein regulatory networks in human, mouse, and chicken systems, placing them at the center of modules enriched for collagen‐containing ECM, cartilage development, and growth factor signaling. Pathway‐specific PPI networks further positioned CCN1/2 at the interface of TGF‐β/SMAD, BMP, FGF, and integrin/FAK signaling, linking canonical chondrogenic transcription factors (e.g., RUNX2 and SMAD3) and matrix genes (e.g., COL2A1, ACAN, and COL1A1) with mechanosensitive Hippo–YAP/TAZ and cytoskeletal components. Together with previous genetic evidence that CCN2 is essential for skeletal development and matrix production, and that CCN1 regulates chondrocyte maturation,^50^ these findings support a model in which CCN1/2 form a conserved “*core CCN axis*” that coordinates ECM synthesis with growth factor and mechanical cues, rather than acting as isolated downstream targets.^18^, ^51^, ^52^

### 4.2 Species‐specific and model‐specific extensions of CCN regulatory networks

Ortholog mapping revealed a structured conservation landscape. Growth factor and cytokine modules, particularly IGF, EGFR, SMAD, collagen‐activated signaling, HIF‐1, ECM–receptor signaling, and collagen biosynthesis showed high human‐ and mouse‐centered conservation indices and the largest Jaccard overlaps, indicating that the core ligand–receptor and ECM–receptor machinery within CCN networks is strongly conserved.^53^, ^54^, ^55^, ^56^ These findings support the view that the core growth factor sensing and transduction machinery controlling chondrogenic differentiation is evolutionarily stable and likely represents a fundamental regulatory scaffold across mammalian lineages.

In contrast, microenvironmental response pathways and cytoskeletal categories were much less conserved, with low Jaccard index values and many species‐specific genes, particularly in hypoxia‐response and mechanosensing subclasses, indicating that these genes are species‐restricted.^57^ This suggests that, beyond a shared HIF‐1 core, human and mouse chondrocytes recruit distinct hypoxia and mechanical stress modules within their CCN networks. This is consistent with differences in developmental stage and biomechanical context between embryonic limb buds and adult‐like MSC chondrogenesis.^58^ ECM‐related categories occupied an intermediate position, with a conserved core supplemented by species‐specific extensions, particularly in the mouse network. Collectively, these observations imply that mouse micromass cultures are well suited for dissecting conserved developmental and growth factor‐driven aspects of CCN function,^37^ whereas human MSC‐ and hiPSC‐based models are preferable for studying CCN‐linked responses to hypoxia, inflammation, and mechanical stress that are especially relevant to adult articular cartilage and OA.

### 4.3 Pathway‐specific network architecture and multi‐functional hubs

Pathway‐focused PPI networks clarify how CCN proteins are wired into key signaling systems that govern chondrogenesis. In the “chondrocyte proliferation” and “cartilage tissue formation” networks, CCN2 and CCN4, or CCN1, CCN2, and CCN4, form hubs that connect PTHLH and BMP/TGF‐β/FGF inputs to collagen‐rich ECM modules and transcriptional regulators such as RUNX2 and SMAD3.^59^, ^60^ Most canonical chondrogenic regulators in these networks are shared human–mouse orthologs, indicating that CCN‐centered signaling orchestrates proliferation and matrix assembly through a broadly conserved mammalian module rather than a simple SOX9–RUNX2 switch. In the TGF‐β pathway network, CCN1 and its ECM partners sit at the interface between a conserved SMAD–receptor core and additional mouse‐specific ligands, co‐receptors, inflammatory mediators, and Hippo components, positioning CCN1 as a candidate modulator of TGF‐β/BMP signal strength in a mechanosensitive context.^51^ In the Hippo network, CCN2 associates with a conserved LATS2–TAZ module that integrates TGF‐β and Wnt inputs, whereas mouse‐specific expansion of upstream kinases, Wnt ligands, and SMAD paralogs provides species‐specific control over proliferative and differentiation outputs.

Within this framework, a small set of multi‐functional orthologs (COL2A1, COL1A1, TGFBR1, SMAD3, SRC, HIF1A, IGF1, SPP1, and CD44) emerged as central hubs that appear across multiple functional categories. Their broad representation in ECM, growth factor, angiogenesis, hypoxia, and adhesion modules suggests that CCN signaling exerts its diverse effects through organizational control of such hubs, which coordinate matrix assembly, growth factor transduction, and microenvironmental sensing. This systems‐level view implies that modulating CCN activity is likely to influence several aspects of chondrocyte biology simultaneously and underscores the importance of considering the wider CCN network context rather than focusing solely on direct CCN targets.

### 4.4 Avian CCN networks and limitations of the chicken model

The chicken CCN regulatory network contained far fewer functionally annotated genes than the mammalian networks, with only 16 chicken genes mapping to ECM, differentiation, or signaling categories. Comparison of six three‐way orthologs (DCN, PENK, SMAD3, SPARC, TGFB3, and THBS1) showed that human and mouse cultures display gradual, sustained upregulation of these genes, whereas chicken micromass cultures exhibit weaker and often transient induction, with early peaks and subsequent decline for SMAD3 and THBS1 and downregulation of PENK. These patterns suggest that, although a conserved core of CCN‐linked matrix and TGF‐β pathway genes is shared across species, the amplitude and timing of their activation in chicken differ from those in mammalian models. The sparse representation of hypoxia, mechanosensing, and several signaling modules may reflect incomplete *Gallus gallus* annotation and/or genuine differences in environmental sensing, and indicates that avian micromass cultures should be used cautiously for preclinical evaluation of CCN‐targeted strategies.

### 4.5 Single‐cell resolution of CCN‐mediated regulation in vitro and in vivo

The single‐cell analysis of hiPSC‐derived chondrogenesis provided cell‐state‐resolved validation of the CCN regulatory framework inferred from bulk data. Clustering identified mesenchymal and chondrocyte populations that progress from early progenitors to mature chondrocytes.^9^ *CCN1* and *CCN2* were the dominant CCN transcripts, and the CCN regulatory network activity peaked in mesenchymal and early chondrocyte clusters around day 14, then declined in mature chondrocytes, supporting a role in commitment and early matrix assembly rather than in fully mature chondrocytes. Ligand–receptor modeling revealed mesenchymal populations as major CCN signal senders, with the CCN1(CYR61)–ITGB1 axis and additional contributions from receptors such as LRP1, SDC4, and ITGA5, as the strongest predicted communication route from mesenchyme to developing chondrocytes. These findings indicate that CCN‐centered signaling is particularly critical during transitional phases of human chondrogenic differentiation, when ECM assembly and fate specification are being established.

By intersecting human and mouse CCN networks to define a homologous CCN regulatory set, we extended these insights to in vivo embryonic limb development. In human embryonic limb single‐cell data, *CCN1*/*2* and *ITGB1* were highly expressed in mesenchymal and chondrocyte populations, whereas *CCN3*, *CCN4*, and *CCN6* showed distinct enrichment in articular chondrocytes, mature osteoblasts, and hypertrophic chondrocytes, respectively. These patterns are largely absent from the in vitro systems. The homologous CCN network was active in proximal mesenchyme and chondroprogenitors, remained elevated in mature and hypertrophic chondrocytes, and peaked in ossification‐stage cell types. In mouse embryonic limbs, *Ccn1* and *Itgb1* showed similar dominance in mesenchyme, cartilage, and growth plate, whereas *Ccn2* expression and CCN network activity were concentrated in Col1a1^+^ mesenchymal cells and growth plate chondrocytes. Together, these in vivo observations show that the CCN1/2‐dominated program identified in vitro is conserved across species, but that late‐stage deployment of CCN networks, particularly in mature and hypertrophic chondrocytes, differs between models. Human embryonic data suggest sustained CCN activity in mature cartilage and pre‐ossification stages, whereas mouse and hiPSC data show relative attenuation in mature chondrocytes followed by renewed activation around the growth plate and ossification.

### 4.6 Strengths, limitations, and future directions

A major strength of this work is the systematic CCN‐focused comparison of multiple chondrogenic models combined with orthogonal single‐cell analyses in both in vitro and in vivo systems. The integrative use of established well‐characterized differentiation systems and publicly accessible datasets also enhances reproducibility and facilitates direct comparison with existing literature on chondrogenesis and OA. Methodologically, the study demonstrates that integrating WGCNA, STRING‐based PPI, ortholog mapping, and single‐cell ligand–receptor inference provides a generalizable strategy for defining “regulatory neighbourhoods” around matricellular proteins and comparing them across species and developmental contexts.

Several limitations should be acknowledged. All bulk datasets are derived from in vitro systems that lack the complex biomechanical, vascular, and immune environment of native joints and growth plates. The CCN‐centered regulatory networks were inferred from co‐expression and literature‐based PPI resources, which are subject to database bias and do not prove direct regulatory interactions. In particular, WGCNA was applied to relatively small sample sets especially in the mouse dataset, below the ideal size for highly stable module detection. Sensitivity analyses indicated only moderate module stability, and the co‐expression relationships between CCN family members and associated genes should therefore be viewed as computational predictions with limited statistical power. Consequently, our study should be regarded as exploratory and primarily descriptive, with the specific molecular mechanisms suggested by the WGCNA framework requiring experimental validation. Ortholog‐based collapsing of genes may also obscure lineage‐specific duplications and functional divergence. Finally, the single‐cell analyses are currently based on one human hiPSC dataset and limited in vivo studies; broader validation in primary human OA cartilage, and additional developmental datasets will be important.

Despite these caveats, the present data refine and strengthen the implications of a conserved CCN1/2‐centered axis for cartilage repair and OA. The expanded CCN regulatory networks show that CCN1/2 are embedded in ECM, TGF‐β/BMP, FGF, IGF, and Hippo modules and connect multi‐functional hubs such as COL2A1, ACAN, TGFBR1, SMAD3, RUNX2, HIF1A, IGF1, SPP1, and CD44, suggesting that temporally tuned CCN activity could be utilized to promote early matrix deposition while avoiding excessive hypertrophy or fibrosis. The positioning of CCN1 at the intersection of SMAD, integrin/FAK, and Hippo components raises the possibility of using CCN1‐responsive mechanosensitive pathways to guide bioreactor loading or scaffold design. Cross‐species comparisons further argue that human stem cell‐based systems are preferable for modeling CCN‐mediated responses to hypoxia, inflammation and mechanical stress relevant to OA, whereas embryonic micromass models are well suited to questions of early morphogenesis.

Within this framework, the limited but distinct engagement of CCN3/4/5/6 in specific functional categories, together with their species‐ and stage‐dependent expression, provides a mechanistic rationale for conflicting reports on their pro‐versus anti‐chondrogenic actions. Our analysis suggests that these family members may be most effectively targeted in narrow temporal windows or particular microenvironmental contexts (e.g., perichondral or osteochondral interfaces) rather than as global modulators. Finally, the constrained and temporally shifted activation of CCN‐associated orthologs in chicken micromass cultures indicates that avian models should be used cautiously for preclinical evaluation of CCN‐targeted therapies. It reinforces the importance of validating candidate CCN‐based interventions in humanized or mammalian systems that recapitulate both the conserved CCN1/2 core and the human‐specific hypoxic and mechanosensitive extensions uncovered here.

Future work should therefore combine this comparative network framework with targeted functional modulation of CCN1/2 and selected multi‐functional hubs (e.g., TGFBR1, SMAD3, RUNX2, HIF1A, IGF1, and CD44) in mouse and human chondrogenic systems, integrated with epigenomic profiling and advanced in vivo models. Applying CCN network signatures to single‐cell datasets from OA cartilage and repair tissues may help identify CCN‐driven subpopulations associated with degeneration or repair. Overall, this cross‐species multi‐scale analysis positions CCN proteins, particularly CCN1 and CCN2, as central organizers of conserved chondrogenic networks while delineating how environmental sensing, mechanotransduction, and specific signaling branches diversify between species and between models. The framework offers mechanistic guidance for selecting appropriate chondrogenic models, for timing CCN‐targeted interventions in regenerative strategies, and for exploiting CCN‐regulated pathways as potential therapeutic entry points in cartilage repair and OA therapy.

## AUTHOR CONTRIBUTIONS

All authors made substantial contributions to discussion of the content, writing of the original outline, and reviewing/editing of the article before submission. All authors approve the final version for publication and agree to be accountable for the accuracy and integrity of the study.

## CONFLICT OF INTEREST STATEMENT

The authors declare no conflicts of interest. The authors wrote this paper within the scope of their academic and research positions. None of the authors has any relationships that could be construed as biased or inappropriate. Funding bodies were not involved in the study design, data collection, analysis, or interpretation. The decision to submit this paper for publication was not influenced by any funding bodies.

## ETHICS STATEMENT

All procedures involving animals were conducted in accordance with the institutional guidelines of Wonkwang University and complied with relevant national regulations for the care and use of laboratory animals. The mouse micromass culture experiments were approved by the Wonkwang University Animal Care and Use Committee (protocol numbers WKU18‐23, WKU19‐09, WKU20‐61). Embryos were obtained from pregnant mice that were humanely sacrificed following institutional standard operating procedures, and all efforts were made to minimize animal suffering and to use the minimum number of animals necessary to achieve scientific objectives.

## Supporting information

Supporting Information S1

Figures S1–S10

Tables S1–S5

## Data Availability

The raw and processed bulk RNA‐seq data generated from mouse limb bud micromass cultures in this study have been deposited in the NCBI Sequence Read Archive under BioProject accession number PRJNA1421121 (http://www.ncbi.nlm.nih.gov/bioproject/1421121). Normalized bulk RNA‐seq data for chicken chondroprogenitor micromass cultures were obtained from previously published datasets (NCBI BioProject accessions PRJNA817177; PRJNA938813). HMSC chondrogenesis bulk RNA‐seq data were downloaded from the gene expression omnibus (GEO accession GSE109503) database. Single‐cell RNA‐seq data from human hiPSC‐derived chondrogenic cultures were obtained from the GEO repository (accession GSE160787). Human embryonic limb single‐cell RNA‐seq data were obtained from previously published, quality‐controlled, and annotated public datasets (Lawrence et al. 2025). Mouse embryonic limb single‐cell data were retrieved from GSE142425 and processed using the same normalization and quality‐control pipeline. All analysis scripts have been made publicly available at: https://github.com/znGer‐cel/species‐Specific‐Roles‐of‐CCN‐Proteins‐in‐Cartilage‐Development.
