# Supplementary material for: Species‐specific roles of cellular communication network proteins in cartilage development: A comparative study using in vitro chondrogenic models
Source: J Cell Commun Signal. 2026 Jun 2;20(2):e70089. doi: 10.1002/ccs3.70089 (PMC13240526; doi:10.1002/ccs3.70089)
Supplement: Supplementary file 1 — Supporting Information S1 [file CCS3-20-e70089-s001.docx]

**SUPPORTING INFORMATION**

The supporting information expands the main findings with validation of murine micromass chondrogenesis, species-specific CCN module assignments, reconstructed CCN-centred interaction networks, pathway-level ortholog analyses, and single-cell views of CCN activity during human chondrogenesis. These materials provide the detailed figures and data tables underlying the comparative network, enrichment, conservation, and cell-state analyses described in the manuscript.

*Supplementary file 1*

**Figure S1.** Alcian blue staining confirms progressive extracellular matrix accumulation and cartilage nodule formation in murine limb bud micromass cultures over the 10-day differentiation period.

**Figure S2.** CCN-containing WGCNA modules in chicken, mouse, and human chondrogenic models, highlighting the distribution of CCN1–CCN6 across culture age–associated eigengene modules.

**Figure S3.** CCN-centred protein–protein interaction networks reconstructed from WGCNA modules in human, mouse, and chicken, showing CCN1/CCN2 as high-degree hubs in each species.

**Figure S4–S8.** Pathway-specific CCN-centred PPI networks for chondrocyte proliferation, cartilage tissue formation and patterning, additional signalling pathways, and mechanotransduction.

**Figure S9.** Temporal dynamics of CCN1/CCN2/CCN3 expression during hiPSC-derived chondrogenesis, showing peak expression and CCN network activity around day 14 at single-cell resolution.

**Figure S10.** Single-cell CCN protein regulatory network scores across hiPSC-derived chondrogenic subpopulations and time points, highlighting highest activity in mesenchymal and early chondrocyte states.

*Supplementary file 2*

**Table S1.** Gene and protein lists comprising the CCN protein regulatory networks in human, mouse, and chicken, derived from CCN-containing WGCNA modules and first-neighbour STRING interactions.

**Table S2.** Summary of GO and KEGG enrichment results for CCN regulatory networks, including counts of enriched terms and associated genes in each functional category and pathway subclass.

**Table S3.** Species-level overview of CCN network functional coverage, reporting the number of CCN-associated genes per functional category in human, mouse, and chicken networks.

**Table S4.** Cross-species conservation metrics for human and mouse CCN regulatory networks, including human-centred and mouse-centred conservation indices and Jaccard overlaps for each pathway.

**Table S5.** Detailed breakdown of shared, human-only, and mouse-only CCN-associated genes for all functional categories and pathway subclasses used in the conservation analysis.
