# Supplementary material for: Species‐specific roles of cellular communication network proteins in cartilage development: A comparative study using in vitro chondrogenic models
Source: J Cell Commun Signal. 2026 Jun 2;20(2):e70089. doi: 10.1002/ccs3.70089 (PMC13240526; doi:10.1002/ccs3.70089)
Supplement: Supplementary file 2 — Figures S1–S10 [file CCS3-20-e70089-s002.pdf]

Supplementary Material – Supplementary File 1

Species-Specific Roles of CCN Proteins in Cartilage Development: A Comparative Study Using *in vitro* Chondrogenic Models

Zhangzheng Wang, Judit Vágó, Roland Takács, Szilárd Póliska, Ee Hyun Kim, Eun-Jung Jin, Satoshi Kubota, Celina G Kleer, Bernard Perbal, and Csaba Matta

This PDF file contains:

Figure S1	Progressive cartilage nodule formation in primary mouse embryonic limb bud-derived micromass cultures between days 1 and 10 of chondrogenic differentiation. Phase-contrast photomicrographs show increasing Alcian blue-positive cartilage matrix deposition from sparse, faintly stained foci at day 1 (**A**) and day 3 (**B**) to numerous, coalescing, intensely stained nodules at day 6 (**C**), culminating in an almost continuous cartilage-like matrix by day 10 (**D**). Cultures were established and stained with 1% Alcian blue in 0.1 N HCl as described in Vago *et al*., 2024 (*Curr Protoc*. 2024 Mar;4(3):e1005). Images were acquired using a Leica phase-contrast microscope at 10× original magnification; scale bar, 200 µm.
Figure S2	Module–trait relationships identified by weighted gene co-expression network analysis (WGCNA) in chicken, mouse, and human chondrogenic models. Heatmaps show Pearson correlation coefficients between module eigengenes (rows, colour-coded by standard WGCNA module colours) and culture time points (columns) for **A**. chicken limb bud micromass cultures, **B**. mouse limb bud micromass cultures, and **C**. human MSC-derived chondrogenic pellets. Each tile displays the correlation value (top) and corresponding P value (bottom), with red indicating positive and green indicating negative correlations with culture age as a chondrogenic trait. Modules containing CCN family genes and showing significant correlation with differentiation time were selected for downstream construction of CCN-centred protein–protein interaction networks and functional enrichment analyses.
Figure S3	CCN-centred protein–protein interaction networks in avian, murine, and human chondrogenic systems. **A**. Network of CCN1–CCN3- and CCN4-associated first-neighbour interactors derived from CCN-containing WGCNA modules in chicken limb bud micromass cultures. **B**. CCN-centred network for mouse limb bud micromass cultures, showing an expanded set of CCN1–CCN6 interactors. **C**. CCN family-centred network for human MSC-derived chondrogenic cultures. For each species, genes belonging to CCN-enriched WGCNA modules were queried in STRING (interaction confidence ≥ 0.4) and visualised in Cytoscape. Node size reflects degree (number of connections), with larger, darker orange nodes

indicating higher connectivity, and edges represent undirected protein–protein associations. These networks define the CCN protein regulatory neighbourhoods used for cross-species ortholog mapping and functional enrichment analyses.

Figures S4–S8 Pathway-resolved CCN-centred protein–protein interaction networks in mammalian chondrogenic cultures. Networks were constructed from the CCN regulatory module by subsetting genes annotated to selected Gene Ontology and KEGG pathways and visualising first-neighbour protein–protein interactions.

- *Figure S4:* ECM-related modules, including **A**. ECM assembly, **B**. Cell–matrix adhesion, and **C**. Collagen biosynthesis.
- *Figure S5:* cell fate and tissue-level processes encompassing **A**. Mesenchymal cell morphogenesis, **B**. Ossification and mineralization, and **C**. Cell–cell adhesion.
- *Figure S6:* networks associated with **A**. Cytoskeleton organisation, **B**. Hypoxic response, **C**. Mechanosensing, **D**. ECM–receptor signalling, and **E**. Rho signalling.
- *Figure S7:* signalling pathway modules, including **A**. SMAD, **B**. BMP, **C**. WNT, **D**. Ras, **E**. MAPK, and **F**. PI3K–Akt signalling.
- *Figure S8:* growth factor and cytokine pathway modules, including **A**. Notch, **B**. FGF, **C**. IGF, **D**. VEGF, **E**. ERBB, **F**. EGFR, and **G**. HIF-1 signalling.

In all panels, nodes represent proteins and edges indicate undirected STRING interactions (confidence ≥ 0.4); green nodes denote conserved human–mouse orthologs, orange nodes mouse-specific components, and pink nodes human-specific genes present only in the cross-species comparison.

Figure S9 Single-cell expression dynamics of CCN family members during human iPSC-derived chondrogenesis. Violin plots show normalized single-cell RNA-seq expression levels of **A**. CYR61 (CCN1), **B**. CTGF (CCN2), and **C**. NOV (CCN3) across mesenchymal and chondrocyte populations (Mesenchyme_1, Mesenchyme_2, Chondrocytes_1, Chondrocytes_2) over the differentiation time course (control progenitors, CP, and days 1, 7, 14, 28, and 42). Each violin represents the distribution of gene expression within a given cell type and time point, with individual dots corresponding to single cells. CYR61 and CTGF show highest expression in mesenchymal and early chondrocyte populations and remain detectable at later stages, whereas NOV expression is low and sparsely detected across all populations, consistent with the bulk RNA-seq and network-level analyses.

Figure S10 CCN protein regulatory network activity across single-cell populations in hiPSC-derived chondrogenesis. Dot plot showing mean AUCell scores for the CCN protein regulatory network in each single-cell cluster from human hiPSC-derived chondrogenic cultures (GSE160787). The colour scale indicates the mean network activity per cluster, and dot size represents the fraction of cells with an AUCell score above the activity threshold. Network activity is highest in mesenchyme_1 and mesenchyme_2, with lower scores in chondrocyte and neurogenic-lineage clusters, indicating that CCN-centred regulatory programs are most prominent in early mesenchymal and early chondrocyte states.

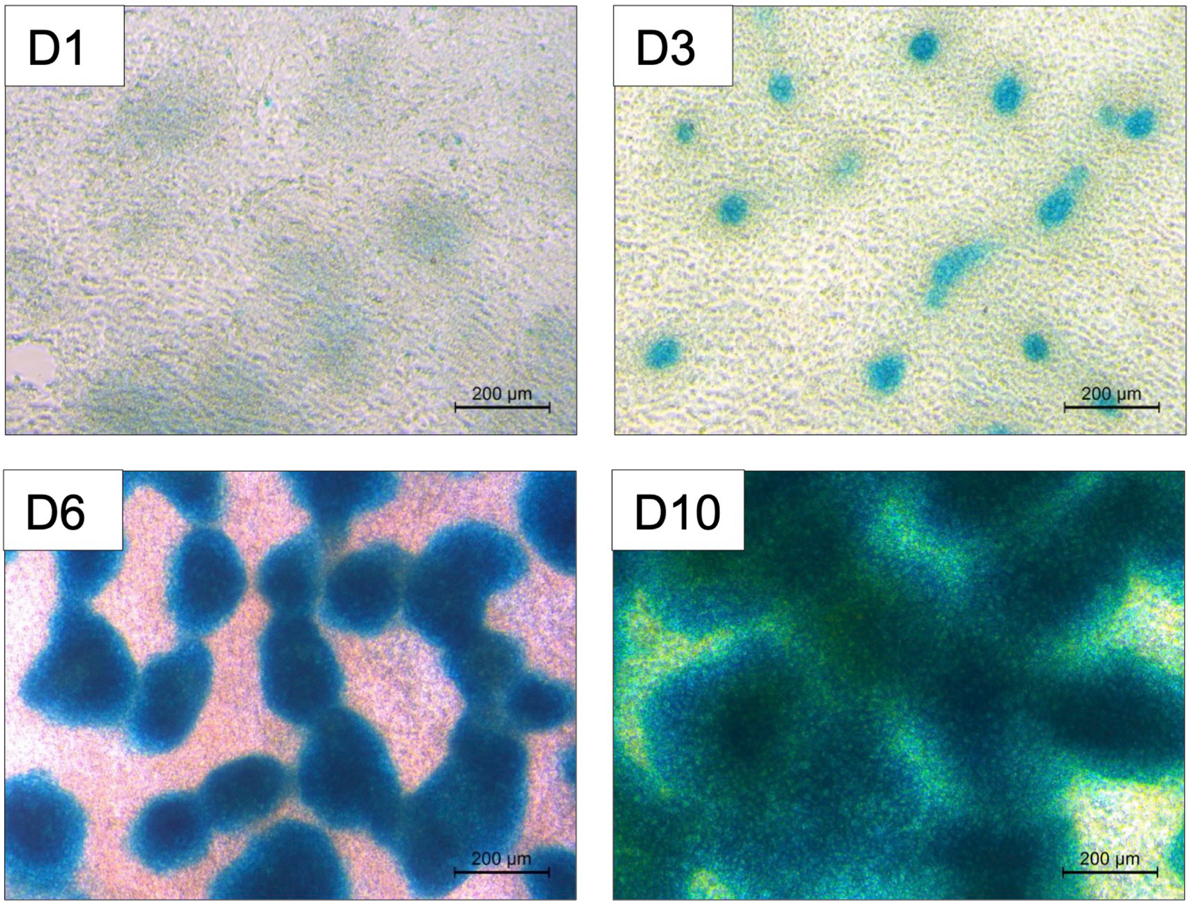

Figure S1. Progressive cartilage nodule formation in primary mouse embryonic limb bud-derived micromass cultures between days 1 and 10 of chondrogenic differentiation. Phase-contrast photomicrographs show increasing Alcian blue-positive cartilage matrix deposition from sparse, faintly stained foci at day 1 (**A**) and day 3 (**B**) to numerous, coalescing, intensely stained nodules at day 6 (**C**), culminating in an almost continuous cartilage-like matrix by day 10 (**D**). Cultures were established and stained with 1% Alcian blue in 0.1 N HCl as described in Vago *et al*., 2024 (*Curr Protoc*. 2024 Mar;4(3):e1005). Images were acquired using a Leica phase-contrast microscope at 10× original magnification; scale bar, 200 µm.

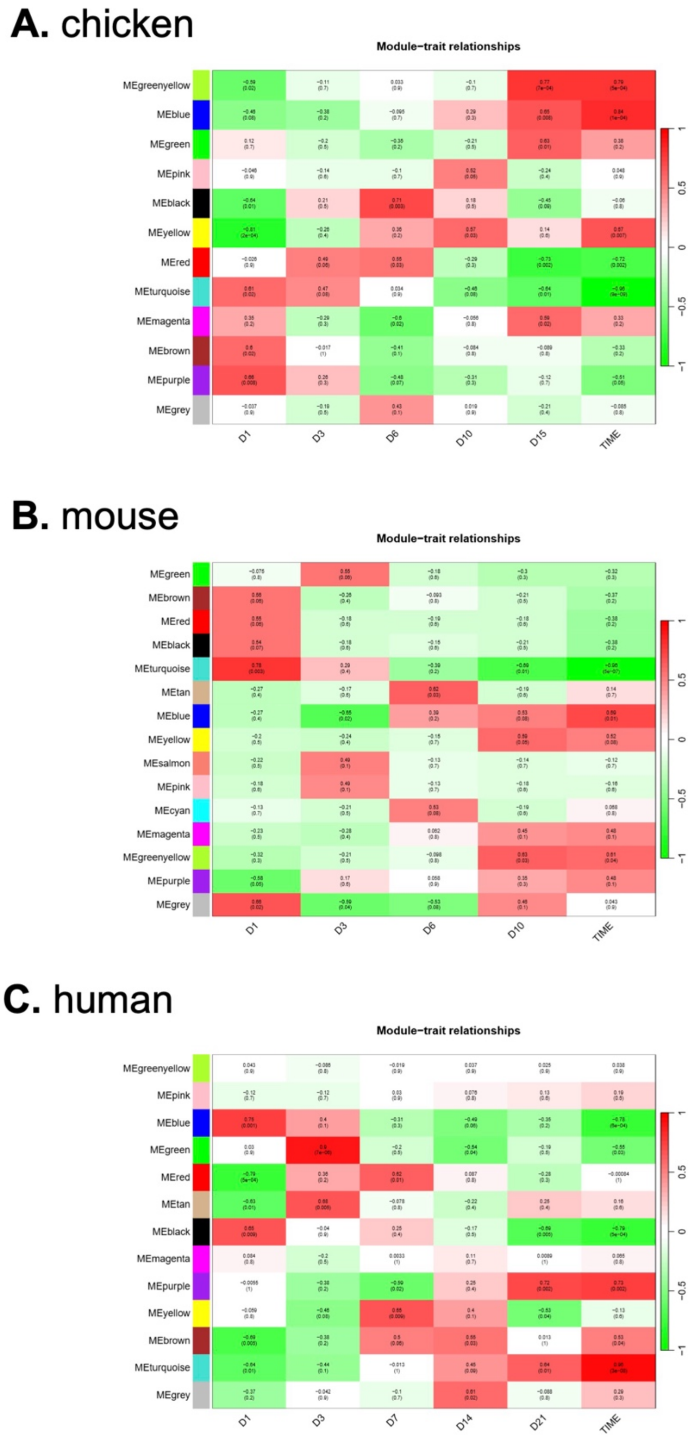

Figure S2. Module–trait relationships identified by weighted gene co-expression network analysis (WGCNA) in chicken, mouse, and human chondrogenic models. Heatmaps show Pearson correlation coefficients between module eigengenes (rows, colour-coded by standard WGCNA module colours) and culture time points (columns) for **A**. chicken limb bud micromass cultures, **B**. mouse limb bud micromass cultures, and **C**. human MSC-derived chondrogenic pellets. Each tile displays the correlation value (top) and corresponding P value (bottom), with red indicating positive and green indicating negative correlations with culture age as a chondrogenic trait. Modules containing CCN family genes and showing significant correlation with differentiation time were selected for downstream construction of CCN-centred protein–protein interaction networks and functional enrichment analyses.

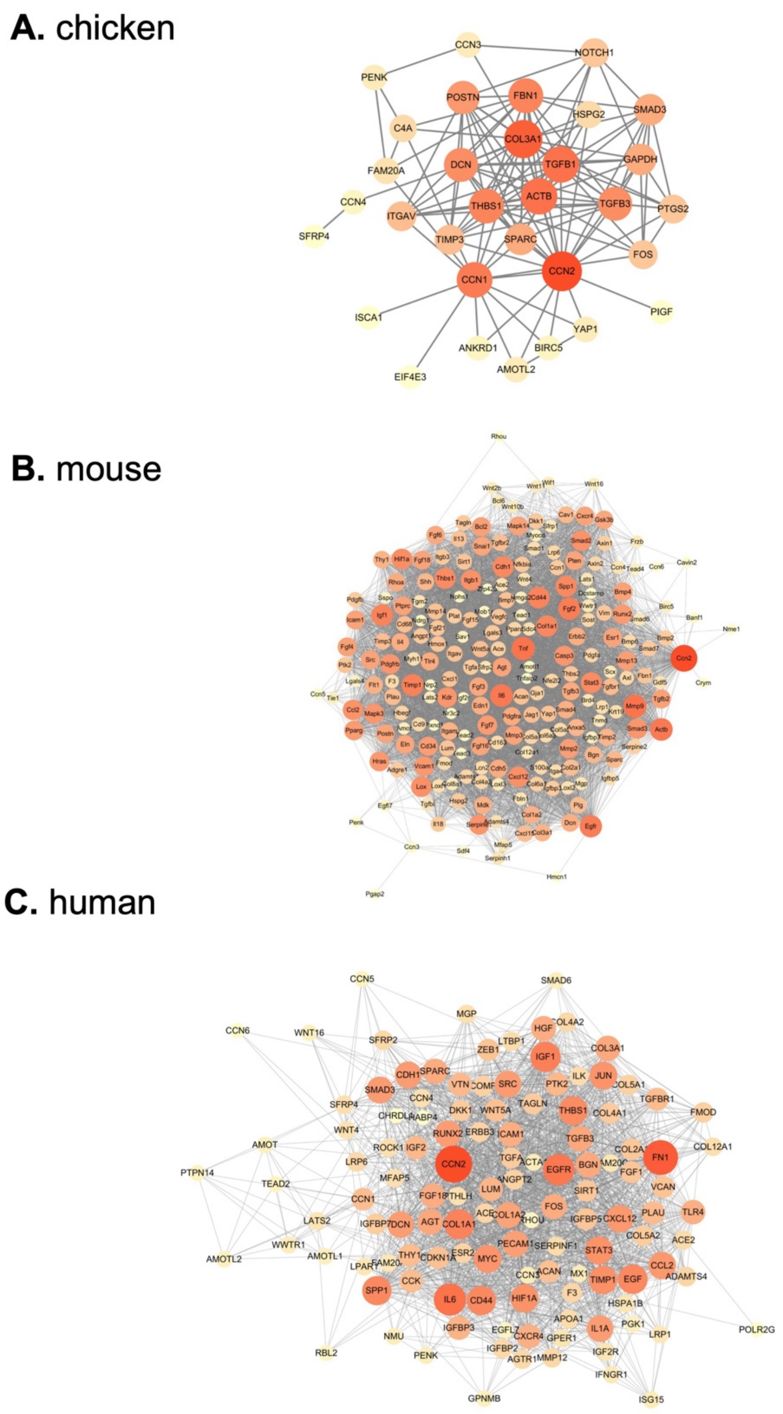

Figure S3. CCN-centred protein–protein interaction networks in avian, murine, and human chondrogenic systems. **A**. Network of CCN1–CCN3- and CCN4-associated first-neighbour interactors derived from CCN-containing WGCNA modules in chicken limb bud micromass cultures. **B**. CCN-centred network for mouse limb bud micromass cultures, showing an expanded set of CCN1–CCN6 interactors. **C**. CCN family-centred network for human MSC-derived chondrogenic cultures. For each species, genes belonging to CCN-enriched WGCNA modules were queried in STRING (interaction confidence ≥ 0.4) and visualised in Cytoscape. Node size reflects degree (number of connections), with larger, darker orange nodes indicating higher connectivity, and edges represent undirected protein–protein associations. These networks define the CCN protein regulatory neighbourhoods used for cross-species ortholog mapping and functional enrichment analyses.

Figure S4.

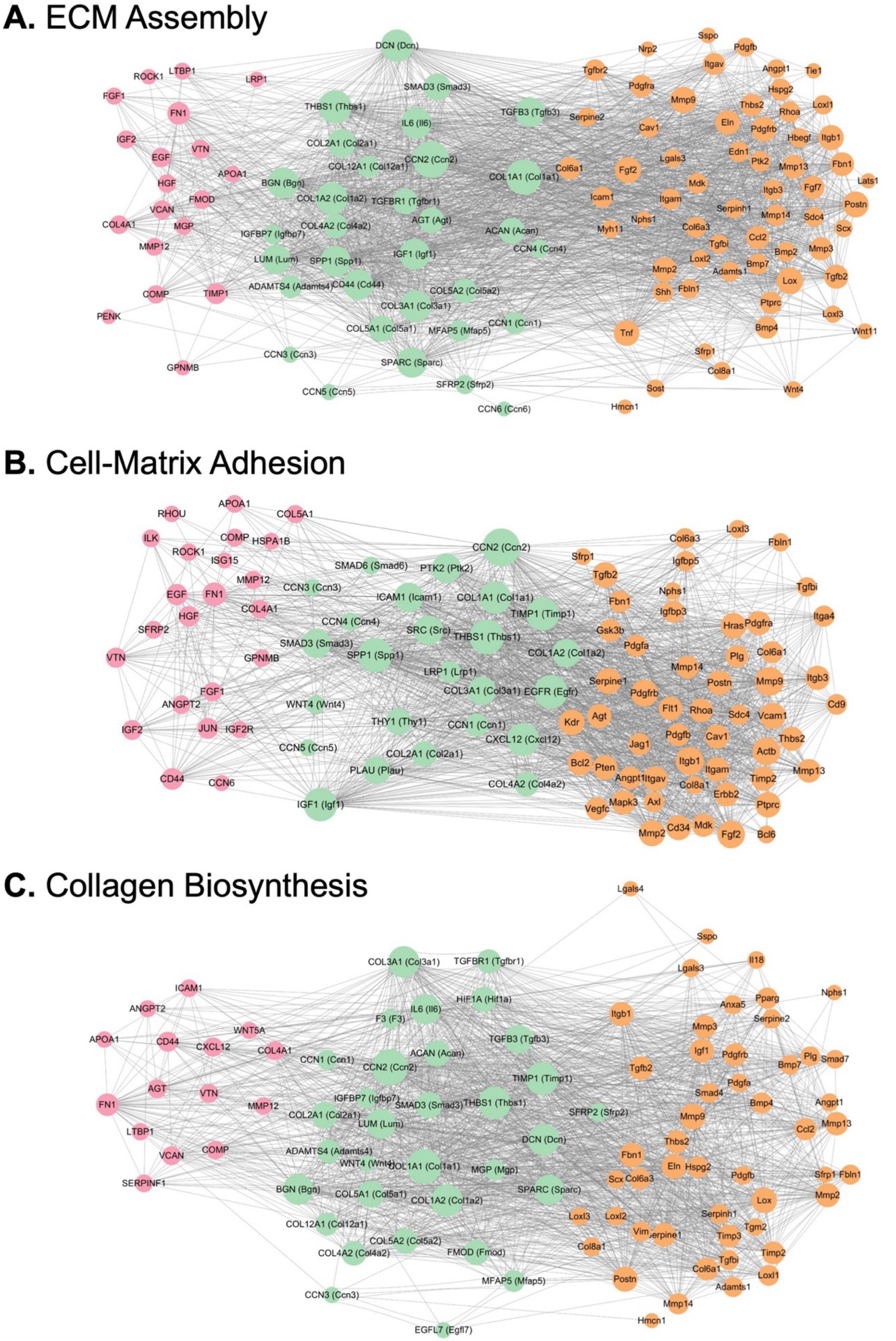
A. ECM Assembly
DCN (Dcn)
Sspo
Nrp2
Pdgfb
LTBP1
ROCK1
LRP1
Itgav
Angpt1
Tie1
Tgfbr2
SMAD3 (Smad3)
Pdgfra
Hspg2
FGF1
Mmp9
Thbs2
Rhoa
Loxl1
FN1
THBS1 (Thbs1)
TGFB3 (Tgfb3)
Serpine2
IL6 (Il6)
Eln
Pdgfrb
Cav1
IGF2
COL2A1 (Col2a1)
Hbegf
Itgb1
VTN
Edn1
CCN2 (Ccn2)
Lgals3
EGF
Ptk2
Mmp13
Fbn1
COL12A1 (Col12a1)
Col6a1
Fgf2
APOA1
COL1A1 (Col1a1)
Lats1
BGN (Bgn)
Mdk
HGF
Itgb3
Fgf7
FMOD
COL1A2 (Col1a2)
TGFBR1 (Tgfbr1)
Icam1
Itgam
Postn
Serpinh1
VCAN
Mmp14
Nphs1
Sdc4
AGT (Agt)
COL4A1
MGP
COL4A2 (Col4a2)
Myh11
Col6a3
Scx
IGFBP7 (Igfbp7)
ACAN (Acan)
Ccl2
Tgfbi
MMP12
CCN4 (Ccn4)
Bmp3
Bmp2
Loxl2
LUM (Lum)
SPP1 (Spp1)
IGF1 (Igf1)
Bmp7
Adamts1
Mmp2
Tgfb2
Lox
ADAMTS4 (Adamts4)
CD44 (Cd44)
COL5A2 (Col5a2)
COMP
TIMP1
Shh
Fbln1
COL3A1 (Col3a1)
Ptprc
PENK
Loxl3
CCN1 (Ccn1)
COL5A1 (Col5a1)
MFAP5 (Mfap5)
Tnf
Bmp4
Wnt11
Sfrp1
CCN3 (Ccn3)
GPNMB
SPARC (Sparc)
Col8a1
SFRP2 (Sfrp2)
Sost
Wnt4
CCN5 (Ccn5)
Hmcn1
CCN6 (Ccn6)
B. Cell-Matrix Adhesion
APOA1
RHOU
COL5A1
Loxl3
ILK
COMP
HSPA1B
CCN2 (Ccn2)
Col6a3
Fbln1
ROCK1
Sfrp1
Igfbp5
ISG15
SMAD6 (Smad6)
PTK2 (Ptk2)
Tgfb2
MMP12
Tgfbi
EGF
FN1
CCN3 (Ccn3)
Nphs1
COL4A1
ICAM1 (Icam1)
COL1A1 (Col1a1)
Fbn1
HGF
Igfbp3
Itga4
TIMP1 (Timp1)
CCN4 (Ccn4)
Hras
Pdgfra
SFRP2
Gsk3b
SRC (Src)
THBS1 (Thbs1)
SMAD3 (Smad3)
Pdgfa
Col6a1
VTN
SPP1 (Spp1)
GPNMB
COL1A2 (Col1a2)
Plg
Mmp14
LRP1 (Lrp1)
Itgb3
Serpine1
Postn
Mmp9
Pdgfrb
COL3A1 (Col3a1)
EGFR (Egfr)
FGF1
Cd9
Flt1
Rhoa
ANGPT2
WNT4 (Wnt4)
Sdc4
Vcam1
Agt
THY1 (Thy1)
IGF2
JUN
IGF2R
Kdr
CCN1 (Ccn1)
Pdgfb
Cav1
Thbs2
CXCL12 (Cxcl12)
Jag1
CCN5 (Ccn5)
Actb
COL2A1 (Col2a1)
Itgb1
Mmp13
Bcl2
Pten
Itgam
PLAU (Plau)
CD44
CCN6
Timp2
Angpt1
Itgav
Col8a1
Erbb2
COL4A2 (Col4a2)
IGF1 (Igf1)
Mapk3
Axl
Ptprc
Vegfc
Mdk
Cd34
Mmp2
Fgf2
Bcl6
C. Collagen Biosynthesis
Lgals4
Sspo
COL3A1 (Col3a1)
TGFBR1 (Tgfbr1)
Il18
Lgals3
ICAM1
Nphs1
HIF1A (Hif1a)
Pparg
IL6 (Il6)
Anxa5
ANGPT2
Itgb1
F3 (F3)
Serpine2
WNT5A
Mmp3
APOA1
CD44
CXCL12
TGFB3 (Tgfb3)
COL4A1
Igf1
Pdgfrb
Plg
Smad7
CCN1 (Ccn1)
ACAN (Acan)
Bmp7
CCN2 (Ccn2)
Tgfb2
TIMP1 (Timp1)
Pdgfa
AGT
Angpt1
VTN
Smad4
FN1
IGFBP7 (Igfbp7)
Bmp4
MMP12
SMAD3 (Smad3)
THBS1 (Thbs1)
SFRP2 (Sfrp2)
Mmp13
COL2A1 (Col2a1)
Mmp9
Ccl2
LUM (Lum)
LTBP1
DCN (Dcn)
Thbs2
ADAMTS4 (Adamts4)
COMP
VCAN
Fbn1
WNT4 (Wnt4)
Eln
Hspg2
COL1A1 (Col1a1)
MGP (Mgp)
Scx
Col6a3
Pdgfb
SERPINF1
Sfrp1
Fbln1
BGN (Bgn)
SPARC (Sparc)
COL5A1 (Col5a1)
COL1A2 (Col1a2)
Lox
Mmp2
Serpinh1
Loxl3
Loxl2
COL12A1 (Col12a1)
Tgm2
Vim
Serpine1
Timp3
COL5A2 (Col5a2)
Col8a1
COL4A2 (Col4a2)
FMOD (Fmod)
Timp2
Tgfbi
Col6a1
Loxl1
MFAP5 (Mfap5)
Postn
Adamts1
CCN3 (Ccn3)
Mmp14
Hmcn1
EGFL7 (Egfl7)

Figure S5.

A. Mesenchymal Cell Morphogenesis

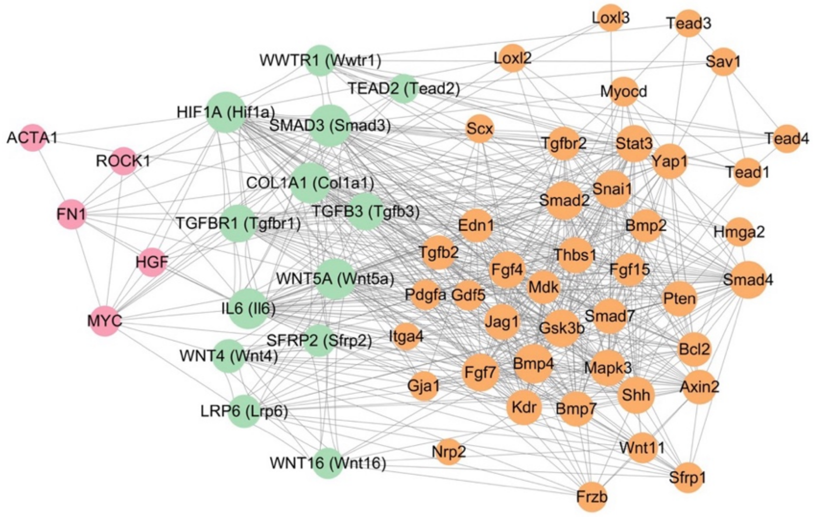

B. Ossification and Mineralization

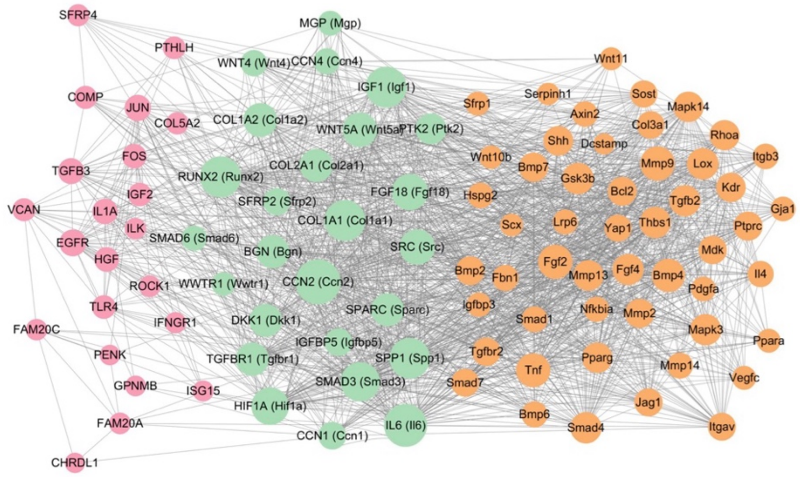

C. Cell-Cell Adhesion

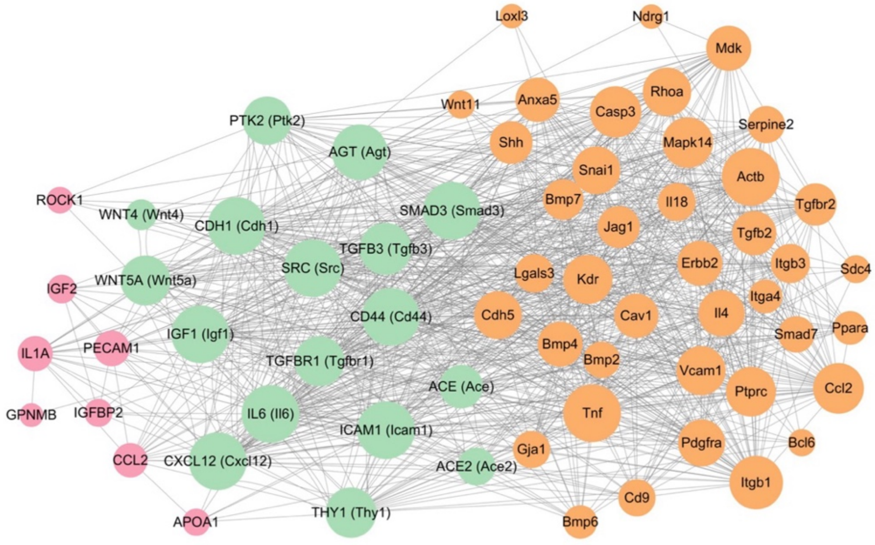

Figure S6.

A. Mesenchymal Cell Morphogenesis

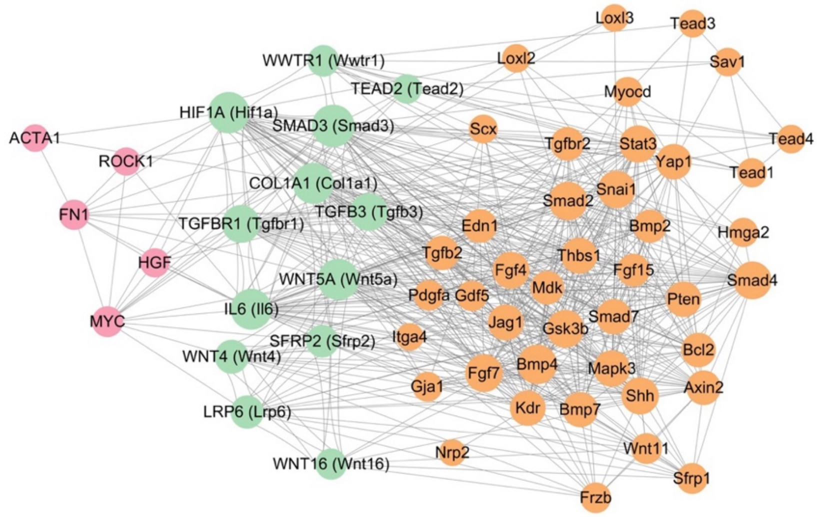

B. Ossification and Mineralization

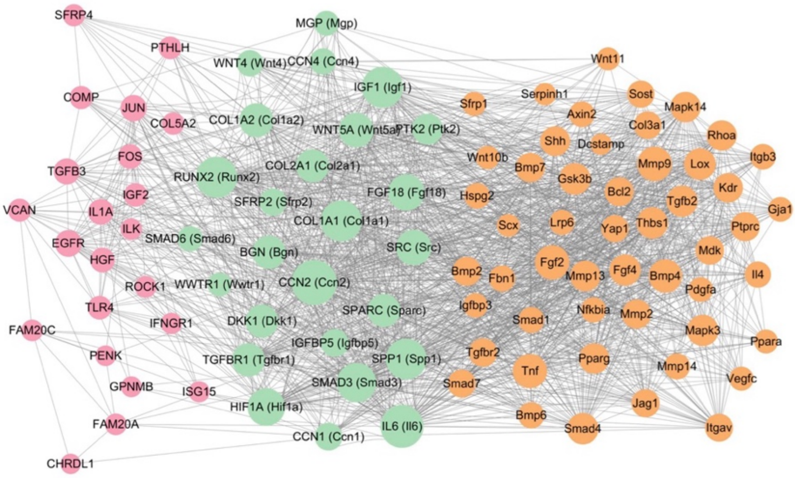

C. Cell-Cell Adhesion

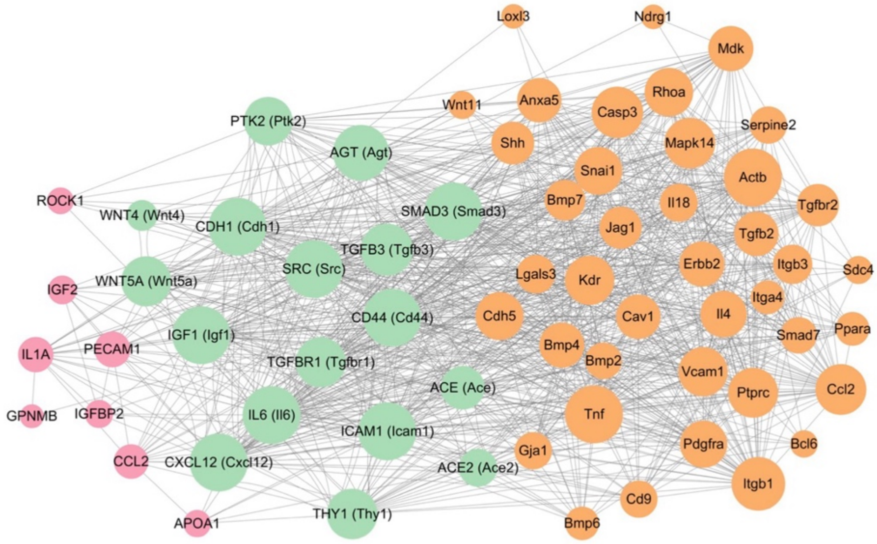

Figure S7.

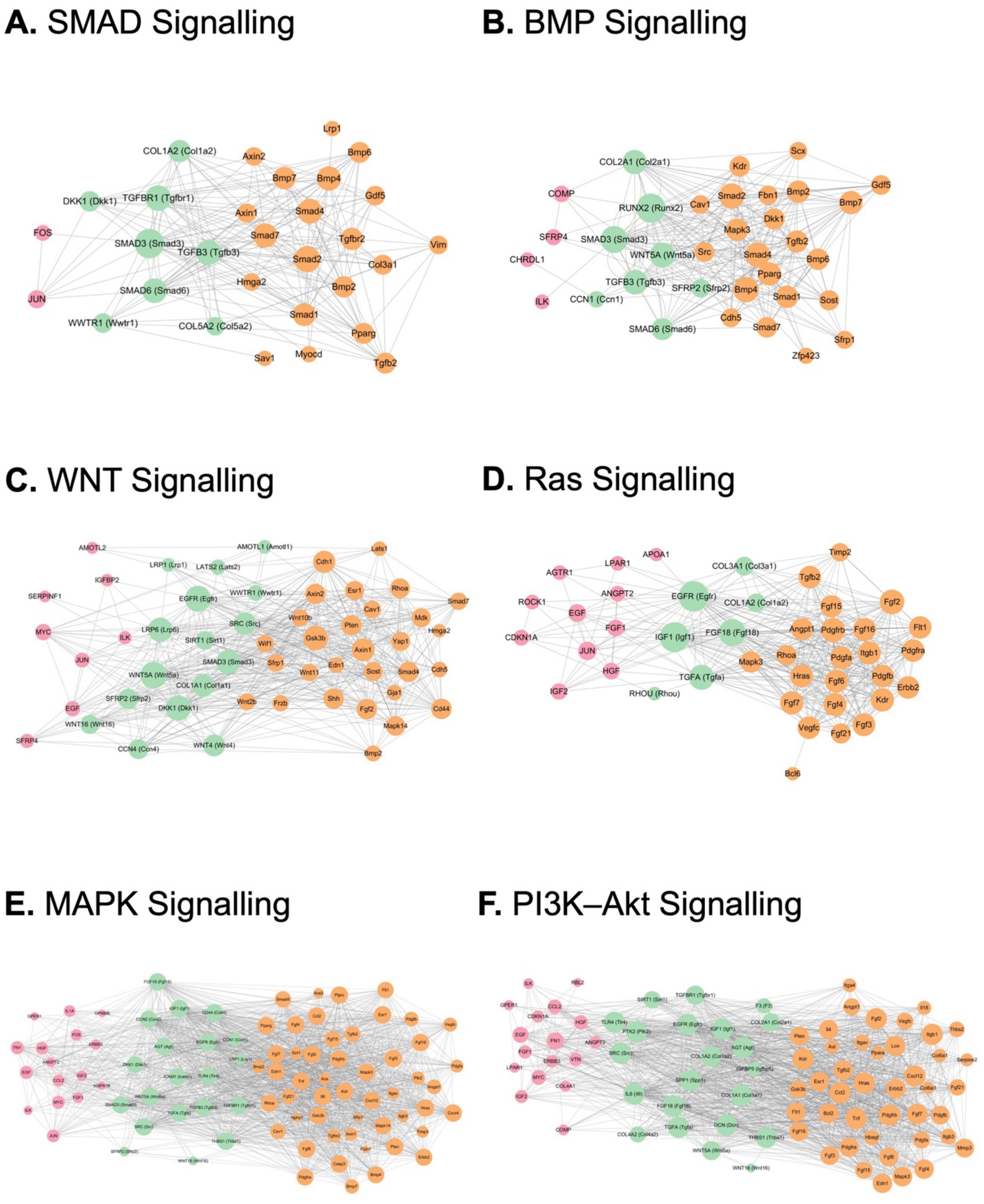

A. SMAD Signalling
COL1A2 (Col1a2)
Lrp1
Axin2
Bmp6
Bmp7
Bmp4
DKK1 (Dkk1)
TGFBR1 (Tgfbr1)
Gdf5
Axin1
Smad4
FOS
SMAD3 (Smad3)
Smad7
TGFB3 (Tgfb3)
Tgfbr2
Vim
Smad2
Col3a1
JUN
SMAD6 (Smad6)
Hmga2
Bmp2
WWTR1 (Wwtr1)
COL5A2 (Col5a2)
Smad1
Pparg
Sav1
Myocd
Tgfb2
B. BMP Signalling
Scx
COL2A1 (Col2a1)
Kdr
Gdf5
COMP
Bmp2
Smad2
Fbn1
Bmp7
RUNX2 (Runx2)
Cav1
Dkk1
SFRP4
SMAD3 (Smad3)
Mapk3
Tgfb2
WNT5A (Wnt5a)
Src
Smad4
Bmp6
CHRDL1
Pparg
TGFB3 (Tgfb3)
SFRP2 (Sfrp2)
Bmp4
Smad1
Sost
ILK
CCN1 (Ccn1)
Cdh5
SMAD6 (Smad6)
Smad7
Sfrp1
Zfp423
C. WNT Signalling
AMOTL2
AMOTL1 (Amotl1)
Lats1
LRP1 (Lrp1)
LATS2 (Lats2)
Cdh1
IGFBP2
SERPINF1
WWTR1 (Wwtr1)
Axin2
Esr1
Rhoa
EGFR (Egfr)
Smad7
Wnt10b
Cav1
Mdk
SRC (Src)
Pten
MYC
LRP6 (Lrp6)
Hmga2
ILK
SIRT1 (Sirt1)
Gsk3b
Wif1
Yap1
JUN
SMAD3 (Smad3)
Axin1
Sfrp1
Edn1
WNT5A (Wnt5a)
Wnt11
Sost
Smad4
Cdh5
COL1A1 (Col1a1)
Gja1
SFRP2 (Sfrp2)
Shh
Wnt2b
Frzb
EGF
DKK1 (Dkk1)
Fgf2
Cd44
WNT16 (Wnt16)
Mapk14
SFRP4
WNT4 (Wnt4)
CCN4 (Ccn4)
Bmp2
D. Ras Signalling
APOA1
Timp2
LPAR1
COL3A1 (Col3a1)
AGTR1
Tgfb2
ANGPT2
EGFR (Egfr)
Fgf2
ROCK1
COL1A2 (Col1a2)
Fgf15
EGF
Flt1
FGF1
Angpt1
Pdgfrb
Fgf16
CDKN1A
IGF1 (Igf1)
FGF18 (Fgf18)
JUN
Pdgfra
Itgb1
Rhoa
Pdgfa
Mapk3
HGF
Hras
TGFA (Tgfa)
Fgf6
Pdgfb
IGF2
RHOU (Rhou)
Erbb2
Kdr
Fgf7
Fgf4
Fgf3
Vegfc
Fgf21
Bcl6
E. MAPK Signalling
F. PI3K–Akt Signalling

Figure S8.

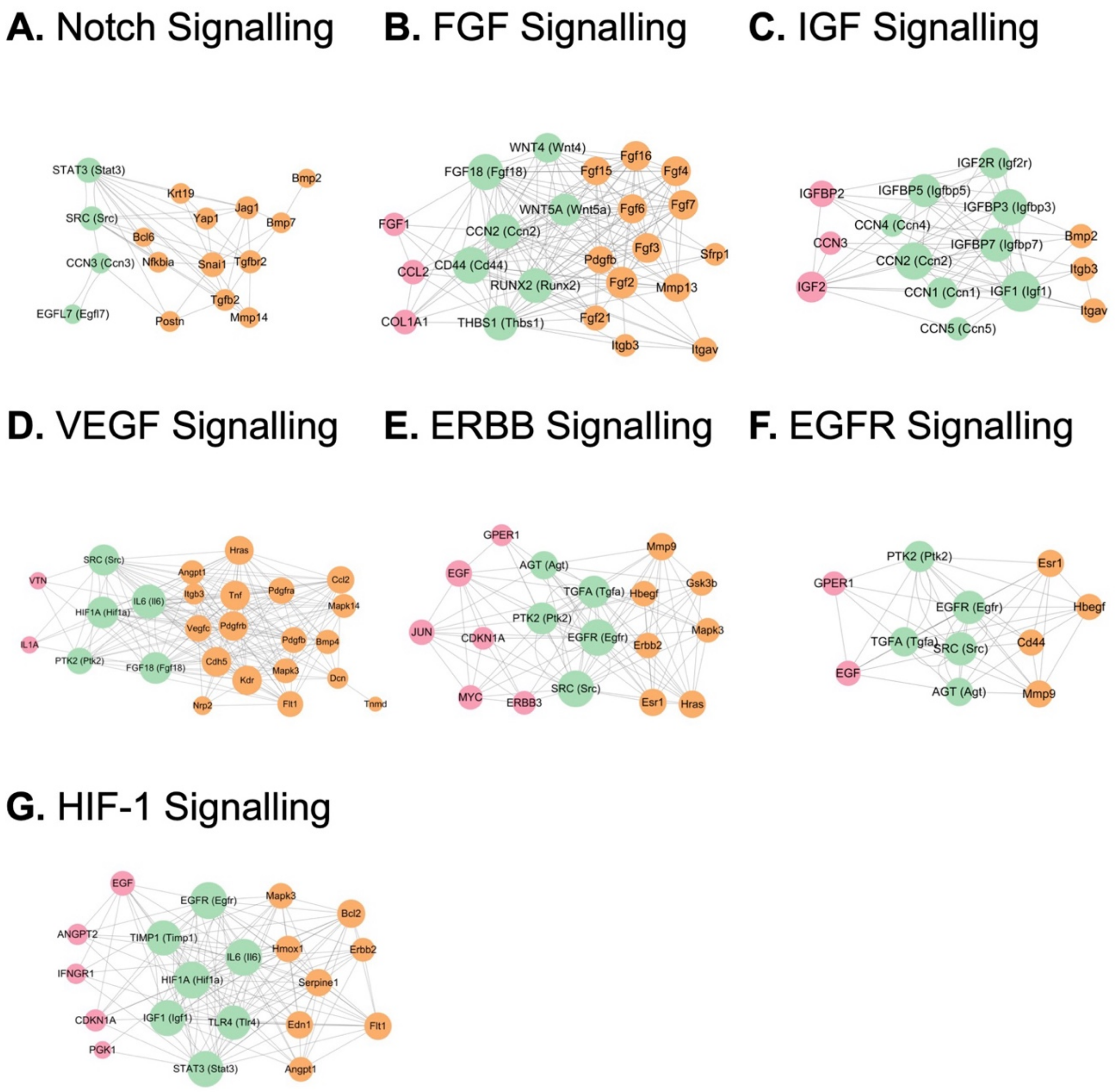

Figures S4–S8. Pathway-resolved CCN-centred protein–protein interaction networks in mammalian chondrogenic cultures. Networks were constructed from the CCN regulatory module by subsetting genes annotated to selected Gene Ontology and KEGG pathways and visualising first-neighbour protein–protein interactions.

- *Figure S4:* ECM-related modules, including **A**. ECM assembly, **B**. Cell–matrix adhesion, and **C**. Collagen biosynthesis.
- *Figure S5:* cell fate and tissue-level processes encompassing **A**. Mesenchymal cell morphogenesis, **B**. Ossification and mineralization, and **C**. Cell–cell adhesion.
- *Figure S6:* networks associated with **A**. Cytoskeleton organisation, **B**. Hypoxic response, **C**. Mechanosensing, **D**. ECM–receptor signalling, and **E**. Rho signalling.
- *Figure S7:* signalling pathway modules, including **A**. SMAD, **B**. BMP, **C**. WNT, **D**. Ras, **E**. MAPK, and **F**. PI3K–Akt signalling.
- *Figure S8:* growth factor and cytokine pathway modules, including **A**. Notch, **B**. FGF, **C**. IGF, **D**. VEGF, **E**. ERBB, **F**. EGFR, and **G**. HIF-1 signalling.

In all panels, nodes represent proteins and edges indicate undirected STRING interactions (confidence ≥ 0.4); green nodes denote conserved human–mouse orthologs, orange nodes mouse-specific components, and pink nodes human-specific genes present only in the cross-species comparison.

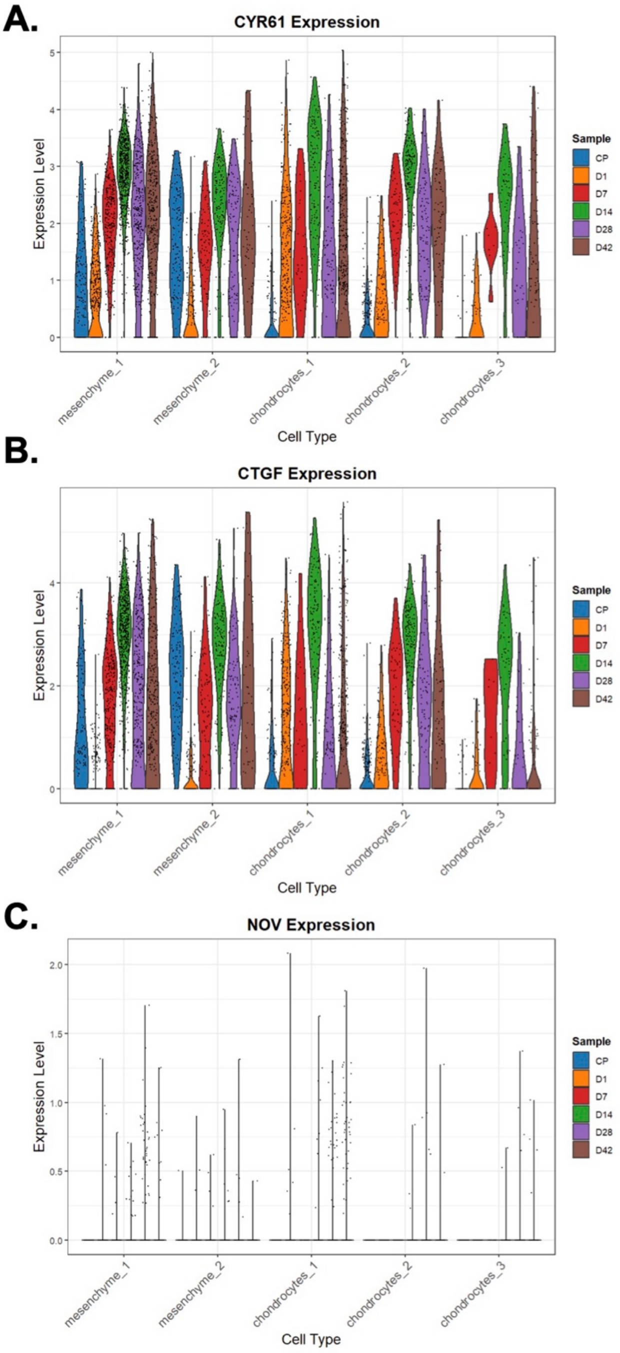

Figure S9. Single-cell expression dynamics of CCN family members during human iPSC-derived chondrogenesis. Violin plots show normalized single-cell RNA-seq expression levels of **A**. CYR61 (CCN1), **B**. CTGF (CCN2), and **C**. NOV (CCN3) across mesenchymal and chondrocyte populations (Mesenchyme_1, Mesenchyme_2, Chondrocytes_1, Chondrocytes_2) over the differentiation time course (control progenitors, CP, and days 1, 7, 14, 28, and 42). Each violin represents the distribution of gene expression within a given cell type and time point, with individual dots corresponding to single cells. CYR61 and CTGF show highest expression in mesenchymal and early chondrocyte populations and remain detectable at later stages, whereas NOV expression is low and sparsely detected across all populations, consistent with the bulk RNA-seq and network-level analyses.

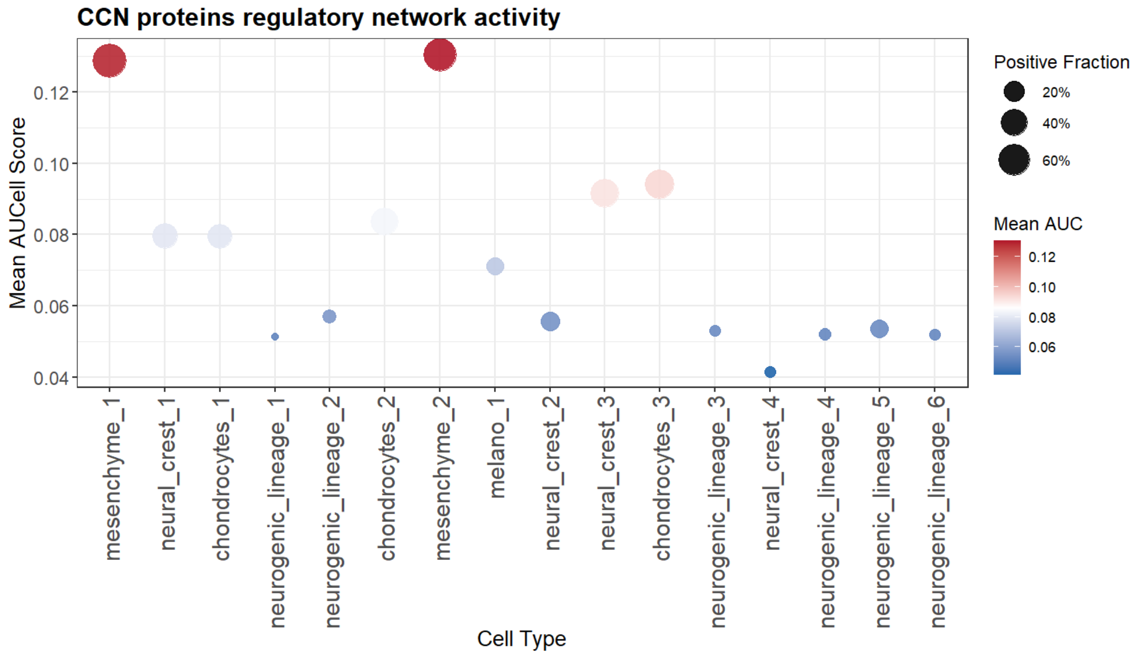

Figure S10. CCN protein regulatory network activity across single-cell populations in hiPSC-derived chondrogenesis. Dot plot showing mean AUCell scores for the CCN protein regulatory network in each single-cell cluster from human hiPSC-derived chondrogenic cultures (GSE160787). The colour scale indicates the mean network activity per cluster, and dot size represents the fraction of cells with an AUCell score above the activity threshold. Network activity is highest in mesenchyme_1 and mesenchyme_2, with lower scores in chondrocyte and neurogenic-lineage clusters, indicating that CCN-centred regulatory programs are most prominent in early mesenchymal and early chondrocyte states.
